# Severe salinity reshapes the ionomic and metabolic profiles of quinoa leaves and seeds in a genotype-dependent manner

**DOI:** 10.3389/fpls.2026.1991117

**Published:** 2026-10-19

**Authors:** Nieves Fernandez-Garcia, Inmaculada Roman-Garcia, Sven-Erick Jacobsen, Enrique Olmos

**Affiliations:** 1 Department of Plant Stress and Plant Pathology, Centro de Edafología y Biología Aplicada del Segura-Consejo Superior de Investigaciones Científicas (CSIC), Murcia, Spain; 2 Quinoa Quality ApS, Regstrup, Denmark

**Keywords:** glycinebetaine, metabolome, nitrogen, quinoa, salinity, seeds

## Abstract

Quinoa (*Chenopodium quinoa* Willd.) is a highly salt-tolerant crop with considerable potential for cultivation in salt-affected areas. However, responses to severe salinity may differ among genotypes and between vegetative and reproductive organs. In this study, five quinoa genotypes, two of Andean origin and three of European origin, were exposed to 400 mM NaCl, and their growth, reproductive performance, ionomic composition, nitrogen allocation, and primary metabolome were evaluated in leaves and seeds. Despite substantial reductions in plant biomass and seed yield, all genotypes completed their life cycle and produced seeds under severe salinity. Growth and yield responses were presominantly genotype-dependent, although the three European genotypes examined showed a higher harvest index than the two Andean genotypes under both control and saline conditions. Salinity induced marked Na^+^ and Cl^−^ accumulation and genotype-dependent changes in K^+^ homeostasis. Among the genotypes examined, seed K^+^ increased in both Andean genotypes but remained unchanged in the three European genotypes, whereas leaf K^+^ was maintained or increased in the European genotypes but decreased or remained unchanged in the Andean genotypes. Salinity also enhanced glycinebetaine accumulation in both leaves and seeds and altered leaf nitrogen distribution, with the magnitude of these responses varying among genotypes. ^1^H-NMR-based metabolomic analysis revealed extensive genotype-dependent changes in primary metabolism. In leaves, proline generally accumulated, whereas several abundant amino acids and TCA-cycle intermediates decreased or showed contrasting responses among genotypes. Seed metabolic profiles also showed marked genotype-dependent responses to severe salinity. Overall, the results indicate that the response of quinoa to 400 mM NaCl involves common responses, including enhanced glycinebetaine accumulation, together with pronounced genotype-dependent adjustments in growth, reproductive performance, ion homeostasis, nitrogen allocation, and primary metabolism.

## 1 Introduction

Soil salinity represents one of the major challenges to sustainable agriculture worldwide. Salinized agricultural areas have steadily increased over recent decades, mainly as a consequence of climate change and unsustainable agricultural practices, which reduce soil porosity and exacerbate soil salinization (Hassani et al., 2021). However, most economically important agricultural crops are glycophytes and therefore require non-saline soils and irrigation water with low electrical conductivity (Adolf et al., 2013). One of the most promising strategies for restoring degraded agricultural soils involves the introduction of novel crops adapted to abiotic stresses (Panta et al., 2014). Halophytes are plants that have evolved to thrive under saline conditions. Over recent decades, considerable attention has been devoted to exploring the potential of halophytes as alternative food crops (Ventura and Sagi, 2013; Panta et al., 2014; Eisa et al., 2017; Grigore and Vicente, 2023). Nevertheless, only a limited number of halophytic species are currently cultivated for food production, accounting for a negligible proportion of global crop production.

Climate change has major implications for global food security. It is argued that agriculture in the 21st century will become saline, severely limiting the use of traditional cereal crops for human caloric intake (Sun et al., 2025). Owing to its exceptional tolerance to salinity and other environmental stresses, quinoa (*Chenopodium quinoa* Willd.) has been proposed by Sun et al. (2025) as a potential next global crop, particularly for agricultural production under increasingly challenging environmental conditions.

Quinoa is an annual herbaceous species belonging to the family Amaranthaceae, formerly classified within the Chenopodiaceae. Quinoa originated in the Andean region of South America and is estimated to have been domesticated approximately 7,000 years ago (Bazile et al., 2016). It is an allotetraploid crop with remarkable environmental adaptability, and its genome has recently been sequenced (Jarvis et al., 2017). At present, more than 3,000 quinoa accessions have been described, many of which are adapted to extreme environmental conditions, including salinity, frost and drought stress (Jarvis et al., 2017). Compared with conventional cereal crops such as wheat, maize and rice, quinoa grains possess notable nutritional advantages and increased stress tolerance. Quinoa grain contains high levels of protein (12-18%), and especially rich in essential amino acids such as lysine and methionine, and providing substantial amounts of minerals, including iron and calcium, vitamins A, B2 and E, and dietary fiber, while exhibiting a low glycemic index (Olmos et al., 2022). Global quinoa production has followed an upward trajectory. According to FAOSTAT, reported global quinoa production increased from approximately 79,000 tonnes in 2010 to approximately 166,000 tonnes in 2024 (FAO, 2025).

In Europe, quinoa cultivation has expanded considerably in recent years. In northern European countries, quinoa genotypes adapted to local climatic conditions have been developed (Jacobsen, 2017). Similarly, in Mediterranean countries such as Spain, production increased from almost negligible levels to approximately 17,000 tonnes in 2021 (Fernandez-García et al., 2023). A major step in the introduction of quinoa into Europe was the development of photoperiod-neutral and early-maturing genotypes (Christiansen et al., 2010; Jacobsen, 2017).

In glycophytic species, irrigation with water containing more than approximately 40 mM NaCl generally causes marked reductions in both plant growth and yield (Munns et al., 2020). In contrast, many halophytic species display improved growth under moderately saline conditions (Flowers and Colmer, 2008). In quinoa, NaCl concentrations of approximately 100 mM have little or no negative effect on vegetative growth or grain yield (Hariadi et al., 2011; Adolf et al., 2013; Iqbal et al., 2019). However, high salinity levels of 400–500 mM NaCl significantly reduce growth and grain yield, also in quinoa (Roman et al., 2020). A major issue in quinoa cultivation in salinized soils and/or under irrigation with water of high electrical conductivity is the effect of salinity not only on vegetative growth and grain yield, but also on grain quality (Koyro and Eisa, 2008; Yousfi et al., 2025). Nevertheless, studies addressing the seed metabolome and ionome under salt stress remain relatively scarce (Toderich et al., 2020). The high salt tolerance of quinoa has mainly been attributed to the presence of epidermal bladder cells on the leaf surface, which accumulate excess foliar sodium and contribute to maintaining the foliar Na^+^/K^+^ ratio (Shabala et al., 2014; Kiani-Pouya et al., 2017). However, it has recently been reported that quinoa mutants lacking bladder cells did not show reduced salinity tolerance (Moog et al., 2022). We have also recently shown that removal of bladder cells did not impair salinity tolerance in quinoa (Olmos et al., 2024). These surprising findings reopen the question of the fundamental mechanisms underlying salinity tolerance in quinoa. It is therefore evident that additional mechanisms must play crucial roles in quinoa salt tolerance (Lopez-Marqués et al., 2020). This is of considerable interest for guiding future quinoa breeding programs, given that the relevance of bladder cells has been strongly emphasized to date.

It is well established that salinity tolerance involves complex mechanisms affecting physiological, biochemical and molecular processes that ultimately induce developmental and morphological modifications (van Zelm et al., 2020). One of the most relevant mechanisms appears to be the regulation of sodium distribution at both cellular and whole-plant levels. Most halophytes tend to transport sodium to aerial tissues, where it can be compartmentalized in the vacuole, thereby avoiding cytoplasmic toxicity and contributing to osmotic adjustment (Flowers et al., 2015). Studies in quinoa show that sodium partitioning at the whole-plant level depends on the genotype studied (Olmos et al., 2024). Thus, a high proportion of genotypes accumulate sodium in the aerial part, although it accumulates mainly in mature leaves. However, other genotypes are able to exclude sodium from the xylem, thereby preventing its transport from the root to the shoot (Adolf et al., 2012). Kiani-Pouya et al. (2020) proposed that genotypes with low bladder-cell density and tolerance to high salinity possess root sodium-exclusion mechanisms, maintaining low sodium contents in leaf tissues.

The accumulation of osmolytes and osmoprotectants in response to salt stress is widely described in the literature (Slama et al., 2015; Singh et al., 2015; Annunziata et al., 2019). The most frequently studied compounds are proline and glycinebetaine (Ashraf and Foolad, 2007). These compounds can accumulate in the cytoplasm, helping to maintain cell turgor and enabling cell expansion under conditions of increased external osmolality. They may also function as osmoprotectants in the cytoplasm and in cellular organelles. Glycinebetaine has been proposed to accumulate in chloroplasts, protecting photosystem II from salinity-induced damage (Huang et al., 2020). However, the role of glycinebetaine in quinoa remains poorly understood. Some studies have reported no or very low levels of glycinebetaine in quinoa (Ruffino et al., 2010; Maqsood et al., 2021), whereas others have reported high concentrations (Morales et al., 2011). Some authors have even suggested that quinoa is the only species within the Amaranthaceae that does not accumulate glycinebetaine (Slama et al., 2015). Conversely, salinity has been observed to induce glycinebetaine accumulation (Jiang et al., 2016), possibly owing to the induction of betaine aldehyde dehydrogenase (BADH), an enzyme involved in the final step of glycinebetaine synthesis in the chloroplast (McNeil et al., 2001). We have recently proposed that glycinebetaine may be a key compound in the adaptation of quinoa to salt stress (Olmos et al., 2024).

However, the literature on the effects of abiotic stress and climatic conditions on quinoa seed proteins and metabolites remains limited (Reguera et al., 2018; Yaqoob et al., 2019; Toderich et al., 2020; Matías et al., 2023; Poza-Viejo et al., 2023). During the grain-filling period, many metabolites are mobilized from source organs such as mature leaves (Smith et al., 2018). These compounds are diverse, but the most frequently accumulated include amino acids, mainly proline, organic acids, sugars, sugar alcohols such as sorbitol, mannitol and myo-inositol, and quaternary ammonium compounds or their derivatives, including glycinebetaine, proline betaine, hydroxyproline betaine and β-alanine betaine (Slama et al., 2015). Many of these compounds can be found in quinoa seeds, although most are synthesized in the leaves and transported to the seeds during grain filling. Therefore, stress conditions may affect the concentrations of compounds relevant to nutritional quality, such as essential amino acids and sugars.

In the present study, we aimed to compare the responses to severe salinity (400 mM NaCl) of quinoa genotypes adapted to northern European conditions, characterized by photoperiod neutrality, and Andean genotypes characterized by high salinity tolerance and short-day photoperiod requirements. Accordingly, the physiological, ionomic, and metabolic responses identified here should be interpreted specifically within the context of severe salinity and cannot be extrapolated to mild or moderate salt stress. Further studies across a broader range of NaCl concentrations will be required to establish dose-dependent responses and determine whether the genotype-specific patterns observed here are maintained at lower salinity levels.

## 2 Materials and methods

### 2.1 Plant material and salt treatments

Five quinoa genotypes of different geographical origins were used: Pandela Rosada (hereafter PR) and Pasankalla (hereafter PSK), of Andean origin, and Puno (hereafter PU), Titicaca (hereafter TI) and Vikinga (hereafter VK), of European origin. The genotypes were selected to compare the responses to severe salinity of traditional Andean germplasm with European-adapted quinoa genotypes. The European genotypes PU, TI and VK were kindly provided by Quinoa Quality (Denmark). Seeds were disinfected by soaking for 10 min in 50% commercial bleach and then rinsed thoroughly with distilled water. They were subsequently germinated in vermiculite in darkness at 26 °C. After germination (2–3 day), seedlings were transferred to a growth chamber under the following conditions: day/night temperature of 25/20 ± 2 °C, relative humidity of 70/90%, and a 16 h photoperiod. After seven days, at the cotyledon stage, seedlings were transplanted into 10 L pots.

Plants were grown in a peat:vermiculite mixture (3:1, v/v) in a temperature-controlled experimental greenhouse at the Agroforestal Experimental Station of the University of Murcia, south-eastern Spain (38.019° N, 1.16° W, 90.30 m a.s.l.). Plants from each genotype and treatment were automatically randomized across experimental positions to minimize potential positional effects. Fifteen plants per genotype were assigned to each treatment. For subsequent analyses, plants were randomly selected from each genotype and treatment, and each selected plant was considered an independent biological replicate. Control plants were irrigated with Hoagland solution, whereas salt-treated plants received Hoagland solution supplemented with NaCl. Salt treatment was initiated when plants had developed four to six leaves, starting at 200 mM NaCl and subsequently increasing the concentration by 50 mM every two days until 400 mM was reached. This concentration was maintained until the end of the experiment. Plants were irrigated daily according to crop water requirements using a surface drip irrigation system to minimize water loss.

### 2.2 Cation, anion, total carbon and total nitrogen analysis

Leaf material was dried in an oven at 65 °C for four days, and samples were ground using a Mixer Mill MM400 (Retsch, Düsseldorf, Germany). Samples consisting of 100 mg of dry material were digested in an HNO_3_:H_2_O_2_ mixture (4:1, v/v) in a Milestone Ultraclave microwave oven at 220 °C for 20 min and subsequently passed through 0.45 μm filters (Sartorius Stedim Minisart RC15). Cation quantification was performed using inductively coupled plasma optical emission spectrometry (ICP-OES; Iris Intrepid II, Thermo Electron Corporation, USA).

Anions were determined from 100 mg of dried and ground leaves. Samples were mixed with 10 mL of bidistilled water and shaken for 30 min. They were then centrifuged at 27,000 × g for 20 min, and the supernatant was passed through 0.45 μm filters. The filtrate was analyzed by ion chromatography using an 850 Professional IC Anion system (Metrohm, Herisau, Switzerland).

Total carbon and nitrogen contents were determined using 100 mg of dried and finely ground leaf material with a TruSpec CN elemental analyzer (LECO Corporation St. Joseph, MI, USA). Samples were oxidized under pure oxygen at 900 °C, and the resulting gases were carried by helium through a reduction reactor at 680 °C. After water removal, CO_2_ and N_2_ were selectively separated and quantified, with carbon detected by infrared detection and nitrogen by thermal conductivity detection. Results were expressed as percentages of sample dry weight.

To assess leaf nitrogen distribution, the relative allocation of nitrogen to glycinebetaine (GB), nitrate (NO_3_^−^), and the remaining nitrogen fraction was estimated from their experimentally determined concentrations. The nitrogen associated with GB and NO_3_^−^ was calculated according to the proportion of nitrogen in their respective molecular formulae and expressed as a percentage of total leaf nitrogen. The remaining nitrogen fraction was calculated by subtracting the contributions of GB-N and NO_3_^−^-N from total nitrogen (%Nt-%(Ngb+NO_3_)). This fraction represents the proportion of leaf nitrogen not accounted for by GB and nitrate.

### 2.3 Analysis of primary metabolism in quinoa leaves and seeds by ^1^H-NMR

Leaf and seed samples were collected, frozen in liquid nitrogen, lyophilized and stored at -80 °C until metabolite extraction. Samples were homogenized using a Retsch MM400 (Retsch GmbH, Haan, Germany).

#### 2.3.1 Metabolite extraction

Fifty milligrams of homogenized and lyophilized material were transferred to 2 mL tubes and extracted with 1 mL of CH_3_OH:H_2_O (1:1, v/v). The mixture was vortexed and kept at 4 °C for 30 min, followed by 3 min of intermittent sonication in an ice bath, consisting of three cycles of 1 min sonication and 1 min pause. After centrifugation at 11,000 × g for 20 min at 4 °C, the methanolic supernatant phase was transferred to 1.5 mL microcentrifuge tubes and dried in a speed vacuum concentrator (Concentrator plus/Vacufuge plus 5305, Eppendorf AG, Hamburg, Germany). Dried samples were resuspended in 800 µL of 100 mM potassium phosphate monobasic buffer (KH_2_PO_4_), pH 6.0, prepared in 100% D_2_O for NMR analysis, containing 0.58 mM TSP-d_4_ internal standard (deuterated trimethylsilylpropionic acid sodium salt) (Van der Sar et al., 2013).

#### 2.3.2 ^1^H-NMR measurements

^1^H-NMR spectra were recorded at 300.1 ± 0.1 K^+^ without rotation using four dummy scans prior to the 32 acquisition scans on a Bruker AVIII HD500 NMR spectrometer (500.13 MHz for 1H) equipped with a 5 mm BroadBand Observe cryogenic probe (Bruker Biospin, Rheinstetten, Germany). The acquisition parameters were as follows: free induction decay size, 64K; spectral width, 12.4345 ppm; receiver gain, 28.5; acquisition time, 2.18 s; relaxation delay, 2 s; and line broadening, 0.50 Hz. A standard one-dimensional NOESY pulse sequence (Bruker 1D, noesypr1d) was used to obtain metabolic profiles of plant extracts. Water suppression was achieved by pre-saturation using irradiation at the water frequency during the recycling and mixing times. During sample processing, noise reduction was performed separately for each spectrum based on multilevel signal spectral deconvolution. Baseline correction was then applied, and interpolation of signal areas was used to complete the processing. This procedure provided a metabolic fingerprint of each sample, offering an overview of the most highly represented metabolites, with chemical shifts (δ) expressed in ppm. The NMR equipment detected the signals and recorded them as frequency versus intensity, generating the acquisition spectrum. ^1^H-NMR spectra were processed, and metabolomic analysis was performed using Chenomx NMR Suite software, version 8.3 (Chenomx, Edmonton, Canada). Samples were processed by calibration against the reference peak of the internal standard TSP-d_4_.

Metabolite levels were calculated as milligrams per gram of freeze-dried plant extract from the least-overlapping NMR signals of metabolites and TSP at known concentration, assuming minor intersample variation in spin-lattice relaxation time for the same protons. These values were expressed as mean ± standard deviation and subjected to classical one-way ANOVA using SPSS 25.0 software, followed by Tukey’s *post hoc* test at p < 0.05.

### 2.4 Statistical and multivariate analysis

A two-way analysis of variance (ANOVA) was performed to assess the effects of genotype (PSK, PR, TI, VK, and PU), salinity treatment (0 and 400 mM NaCl), and their interaction (genotype × salinity) on phenotypic, ionomic, and metabolic parameters. Tukey’s test was applied as a post-ANOVA test at a 5% significance level. Plant height increment was determined using all 15 plants available for each genotype × treatment combination. For all other measurements and analyses, five plants were randomly selected from the 15 plants within each genotype × treatment combination. Each selected plant was analyzed individually and considered an independent biological replicate. The number of biological replicates used for each analysis is indicated in the corresponding figures and tables. Statistical analyses were performed using Statistix software, version 8.1.

For multivariate statistical analysis, partial least squares–discriminant analysis (PLS-DA) was performed using the web-based tool MetaboAnalyst 6.0. Variable importance in projection (VIP) scores were calculated to assess the contribution of each metabolite to group discrimination, and hierarchical clustering heatmaps were generated to visualize differences in metabolite profiles among experimental groups. PLS-DA model performance was evaluated using 5-fold cross-validation, and four components were retained for the final models based on predictive ability (Q²). Model robustness and statistical significance were further assessed by permutation testing using 1,000 random permutations of the class labels. Model goodness of fit and predictive ability were evaluated using R² and Q², respectively.

## 3 Results

### 3.1 High salinity affects plant performance and grain physical parameters, and reduces grain yield

Plant dry weight was significantly reduced in all genotypes (**Table 1**). The greatest reduction in dry weight was observed in TI (61.4%), whereas the lowest reduction was recorded in VK (35.5%). Analysis of the effect of salinity on plant height growth revealed that all genotypes exhibited a significant reduction, with the greatest decrease observed in the VK genotype (**Supplementary Figure S1**). Similarly, seed yield per plant was significantly reduced in all genotypes. The European genotypes showed the highest seed yield per plant under both control and saline conditions. However, the greatest reduction in seed yield was observed in VK (36%), whereas the lowest reduction was observed in PR (16.5%). In addition, the European genotypes showed a higher harvest index (HI) than the andean genotypes under both control and saline conditions. Salinity significantly increased HI in all genotypes except VK, in which no change was observed (**Table 1**).

**Table 1:** Effect of salinity on plant dry weight (DW), seed yield and harvest index in five quinoa genotypes.

Genotype	Treatment	Plant DW (g)	Seed yield (g plant^-1^)	*Harvested index (%)*
PR	C	65,59 a	27,06 a	0,292 b
	400 mM NaCl	32,99 b	22,59 b	0,406 a
Vk	C	57,33 a	43,74 a	0,433 a
	400 mM NaCl	36,94 b	28,04 b	0,432 a
PSK	C	56,00 a	30,00 a	0,349 b
	400 mM NaCl	32,33 b	24,38 b	0,430 a
TI	C	52,28 a	48,75 a	0,483 b
	400 mM NaCl	20,20 b	40,09 b	0,665 a
PU	C	61,64 a	56,32 a	0,477 b
	400 mM NaCl	31,49 b	40,63 b	0,563 a

Plants were grown under control conditions (C) or subjected to 400 mM NaCl. Data are presented as mean ± SD (n = 5). Different letters indicate significant differences among treatments according to Tukey’s multiple comparison test (P < 0.05).

Two-way ANOVA revealed a highly significant genotype x treatment interaction (P < 0.001) for plant dry weight, seed yield per plant, and harvest index, indicating that the magnitude of the response to salinity differed among genotypes for all three traits (**Supplementary Table S1**).

### 3.2 Seed parameters

Data analysis revealed a genotype-dependent effect of salinity on seed traits (**Table 2**). Salt treatment significantly reduced seed length in all genotypes except TI. The greatest reduction was observed in PR, with a decrease of 13%. Seed weight decreased most markedly in PR (23.7%), PSK (17%) and PU (15%), whereas it was not significantly reduced in VK (7%) or TI (7%). Seed dry weight was not significantly altered in PR, VK or PU, but decreased in PSK and TI. Two-way ANOVA revealed a highly significant genotype x treatment interaction (P < 0.001) for plant seed length, seed weight, and % dry weight, supporting a genotype-dependent effect of severe salinity on these traits (**Supplementary Table S2**).

**Table 2:** Effect of salinity on seed length, 100 seed weight and seed dry matter content in five quinoa genotypes.

	Treatment	Seed length(mm)	100 seed weight(g)	% Dry weigth
PR	C	2,29 a	0,316 a	92,74 a
	400 mM NaCl	1,99 b	0,241 b	92,50 a
VK	C	1,16 a	0,245 a	91,96 a
	400 mM NaCl	1,07 b	0,228 a	92,00 a
PSK	C	2,02 a	0,218 a	92,84 a
	400 mM NaCl	1,92 b	0,181 b	92,42 b
TI	C	2,06 a	0,272 a	92,20 a
	400 mM NaCl	2,06 a	0,254 a	91,52 b
PU	C	1,83 a	0,218 a	91,72 a
	400 mM NaCl	1,77 b	0,185 b	91,74 a

Plants were grown under control conditions (C) or subjected to 400 mM NaCl. Data are presented as mean ± SD (n = 5). Different letters indicate significant differences among treatments according to Tukey’s multiple comparison test (P < 0.05).

### 3.3 Mineral composition of quinoa seeds and leaves under high-salinity conditions

Seed sodium concentration increased significantly in all genotypes under salt treatment (**Figure 1**). PR showed a sodium concentration more than twice that of the other genotypes. Increased potassium concentration was observed in the andean genotypes PSK and PR, whereas potassium remained unchanged in the European genotypes PU, TI and VK (**Figure 1**). The effect of salinity on macronutrient composition differed among genotypes (**Figure 1**). Thus, PSK showed a significant increase in all analyzed elements (Ca^2+^, Mg^2+^, P and S), whereas PR showed only a significant increase in calcium. VK showed only a significant increase in phosphorus concentration, whereas the other macronutrients remained unchanged. In contrast, TI showed significant decreases in Ca^2+^ and S concentrations, whereas Mg^2+^ and P remained unchanged. PU showed a significant decrease in Ca^2+^ concentration, whereas P increased significantly; Mg^2+^ and S concentrations were not altered. The analysis of micronutrient concentrations revealed significant differences between control and salt-treated plants, depending on the genotype (**Figure 2A**). Fe^2+^ concentrations decreased significantly in PR and VK, while no significant changes were observed in the other genotypes. Mn^2+^ concentrations increased significantly in PSK, VK, and PU, decreased in TI, and remained unchanged in PR. Regarding Zn^2+^, salinity significantly increased its concentration in PSK and PU, whereas it decreased in TI and PR and remained unchanged in VK. Cu^2+^ concentrations were the least affected by salinity, with no significant changes observed in any genotype except PSK, where a significant increase was detected.

**Figure 1:**
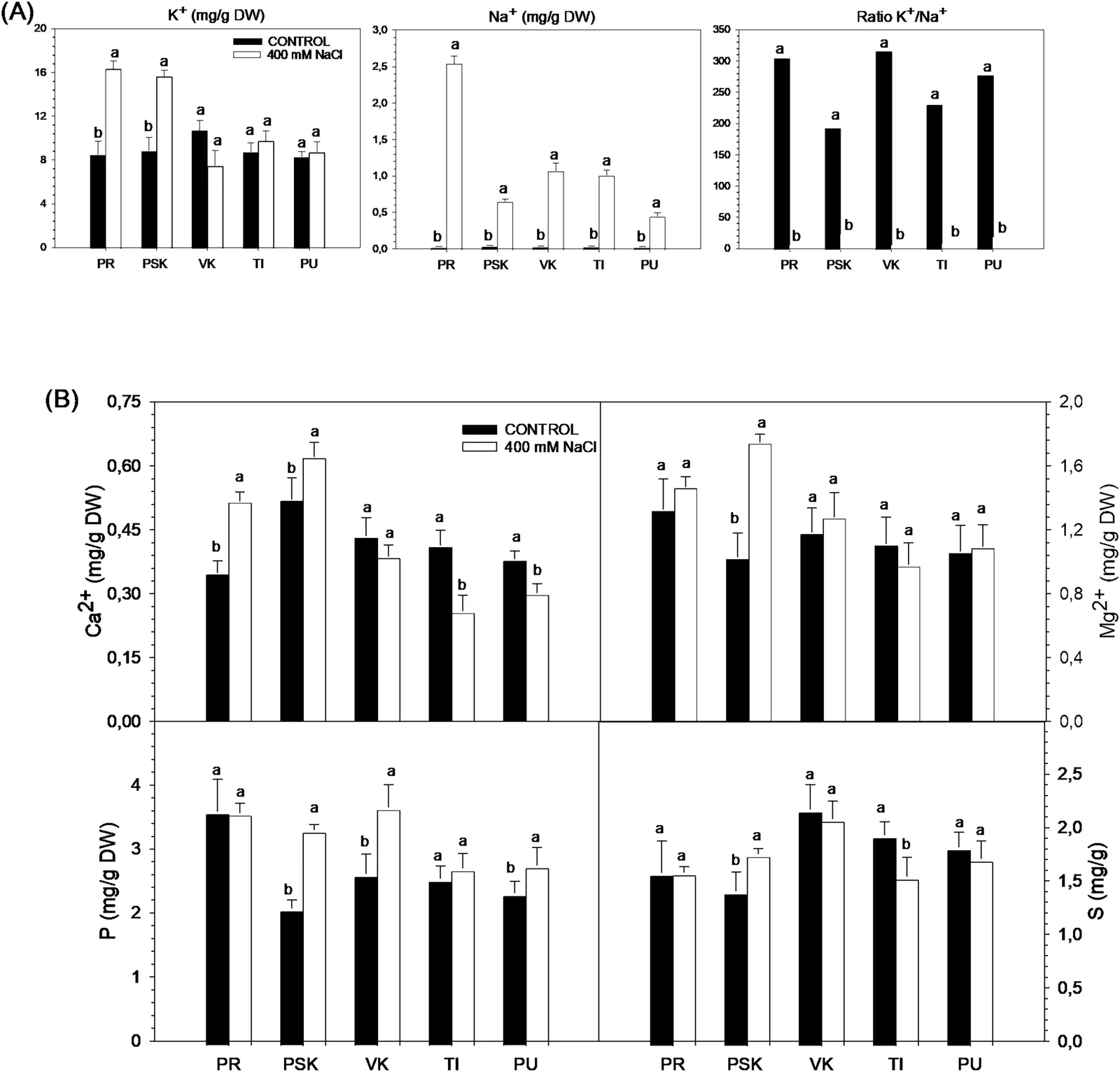
Effect of salinity on the mineral composition of seeds from five quinoa genotypes grown under control conditions or subjected to 400 mM NaCl. **(A)** Sodium (Na^+^) and potassium (K^+^) contents and K^+^/Na^+^ ratio. **(B)** Calcium (Ca^2+^), magnesium (Mg^2+^), phosphorus (P) and sulphur (S) contents. Data are presented as means ± SD (n = 5). Different letters indicate significant differences according to Tukey’s multiple comparison test (P < 0.05).

**Figure 2:**
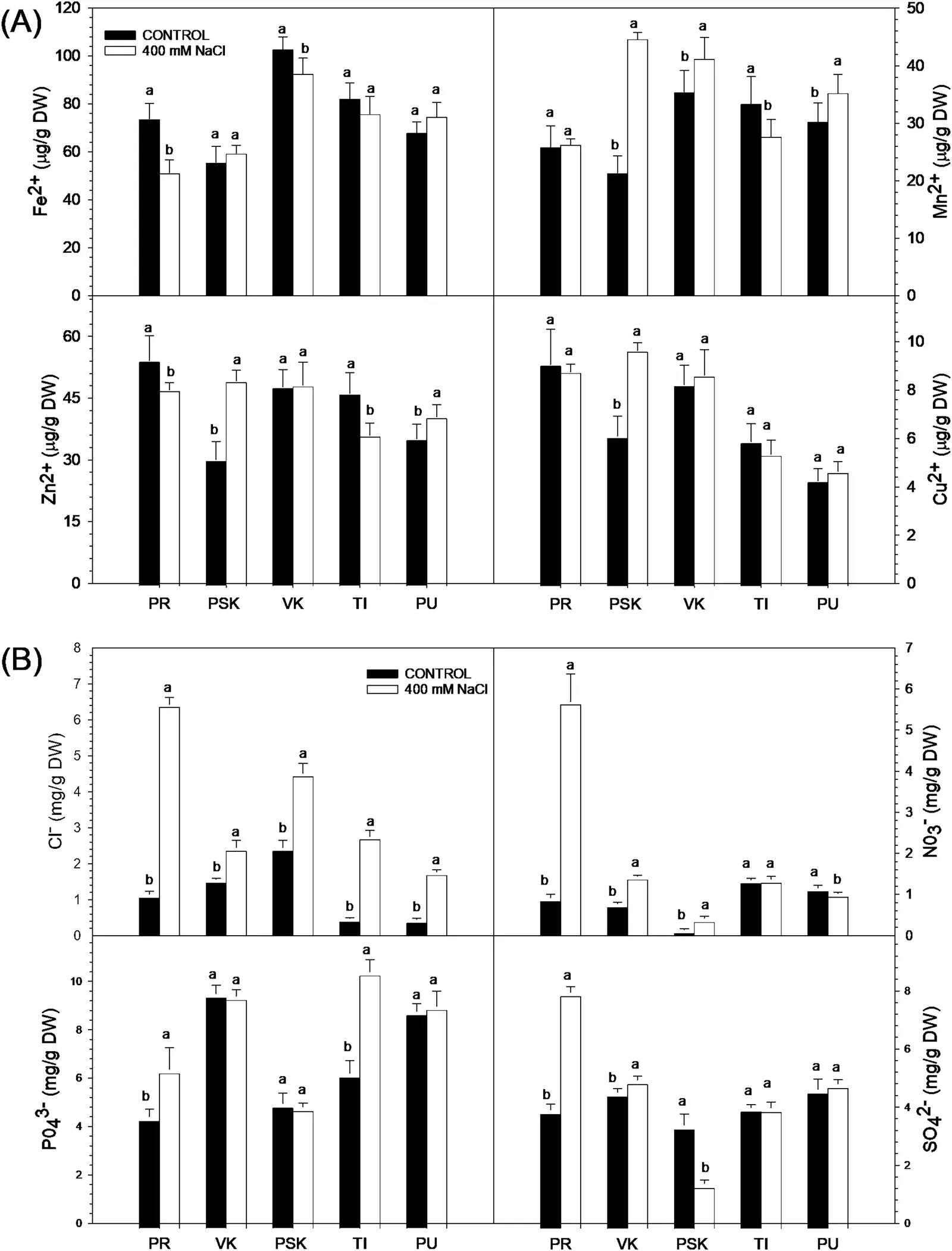
Effect of salinity on mineral composition in seeds of five quinoa genotypes grown under control and 400 mM NaCl conditions. **(A)** Iron (Fe^2+^), manganese (Mn^2+^), zinc (Zn^2+^) and copper (Cu^2+^) contents. **(B)** Chloride (Cl^−^), nitrate (NO_3_^−^), phosphate (PO_4_^3−^) and sulphate (SO_4_^2−^) contents. Data are presented as mean ± SD (n = 5). Different letters indicate significant differences among treatments according to Tukey’s multiple comparison test (P < 0.05).

The concentrations of the major anions were also analyzed (**Figure 2B**). Chloride concentrations increased significantly in all genotypes, with PR showing the most pronounced increase, reaching nearly sevenfold the control value. Nitrate concentrations increased significantly in PR, PSK, and VK, with the greatest increase observed in PR. In contrast, nitrate concentration decreased significantly in PU and remained unchanged in TI. Phosphate concentrations increased significantly in PR and TI, while no significant changes were observed in the other genotypes. Sulphate concentrations increased significantly in PR and VK, with a markedly greater increase in PR. Salinity had no significant effect on sulphate concentrations in TI and PU, whereas a significant decrease was observed in PSK.

Given the metabolic relevance of total nitrogen and carbon contents and their C/N ratio, these parameters were also analyzed (**Figure 3**). Total nitrogen concentration decreased significantly under salinity in PR, PSK, and VK, remained unchanged in PU, and increased significantly in TI. Total carbon content increased significantly in PR, PSK, and TI in response to salinity, remained unchanged in VK, and increased in PU. The C/N ratio increased significantly in PR, PSK, and VK, remained unchanged in PU, and decreased significantly in TI.

**Figure 3:**
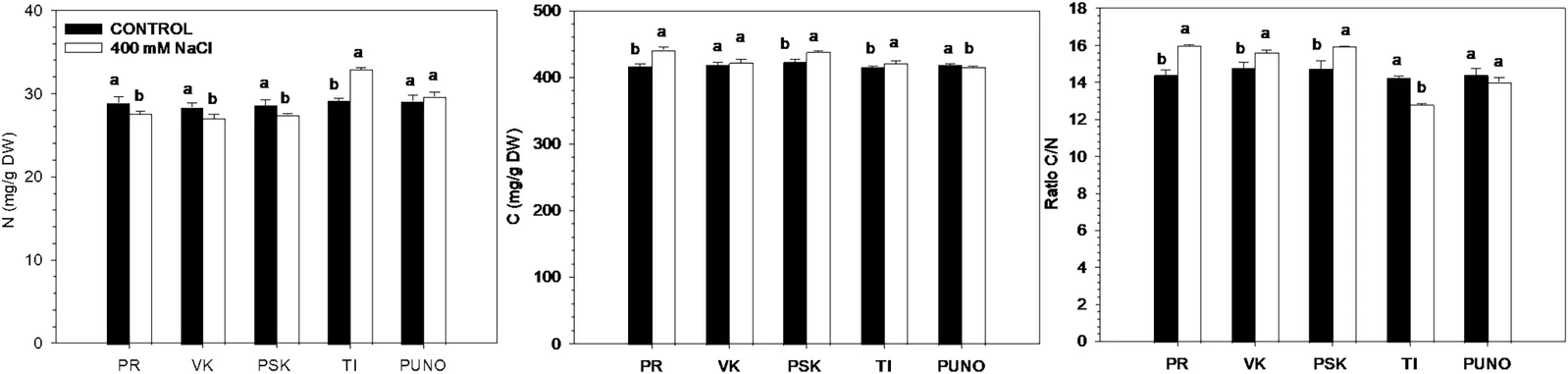
Effect of salinity on Carbon (C), Nitrogen (N) and the ratio C/N in seeds of five quinoa genotypes grown in 400 mM of NaCl. Data are shown in mean ± SD. Different letters indicate the presence of significant differences (P < 0.05) among treatments according to Tukey’s test (n = 5).

Finally, two-way ANOVA revealed a significant genotype × treatment interaction for all seed parameters analyzed, including cations, anions, and carbon and nitrogen contents (**Supplementary Tables S3–S7**).

Leaf Na^+^ concentration increased significantly in all genotypes under salt treatment (**Figure 4A**). K^+^ concentration under salt treatment was significantly reduced in PR but was not altered in PSK. The European genotypes PU and TI showed a significant increase in K^+^ concentration, whereas VK showed no change.

**Figure 4:**
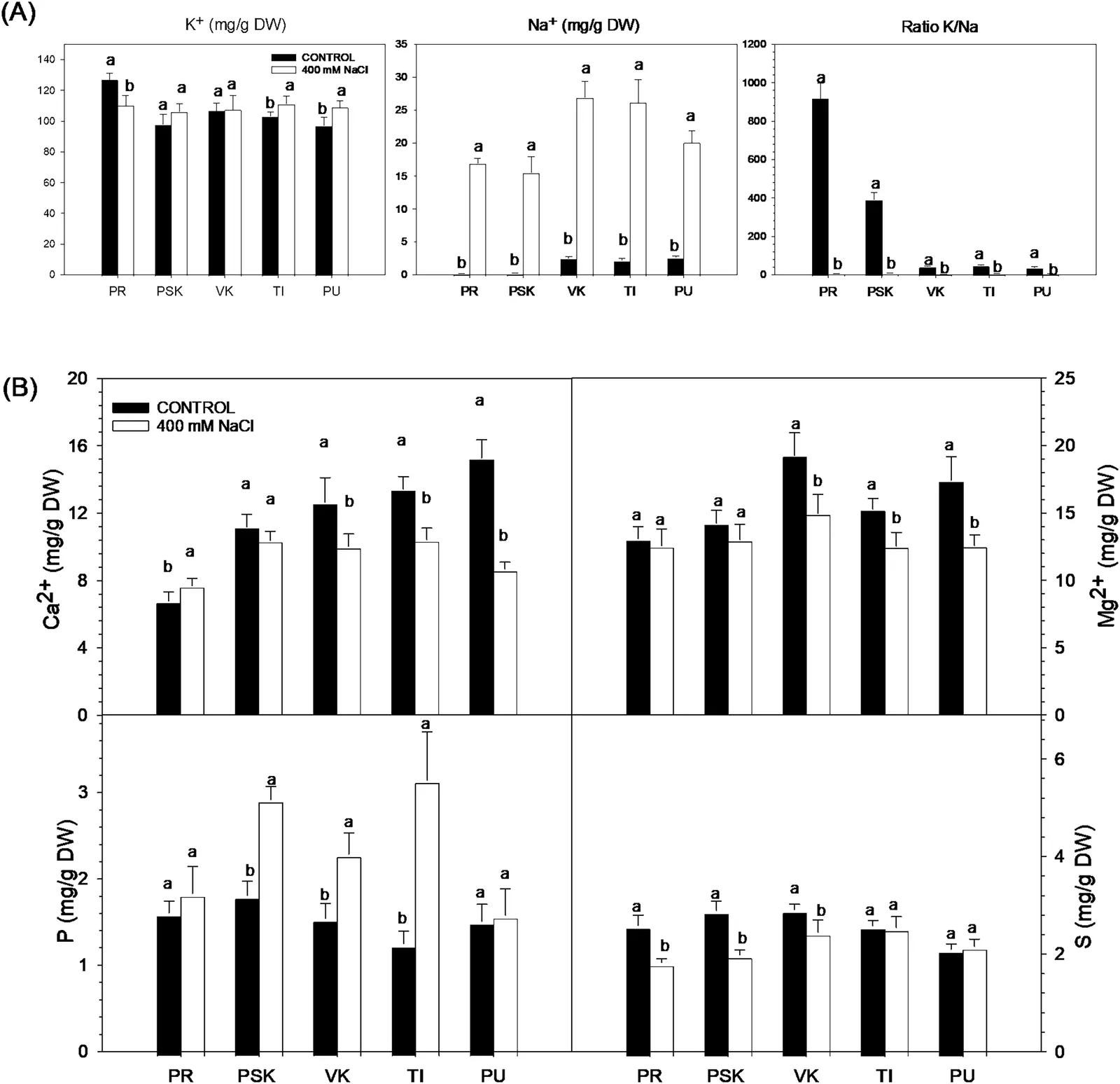
Effect of salinity on the mineral composition of leaves from five quinoa genotypes grown under control conditions or subjected to 400 mM NaCl. **(A)** Sodium (Na^+^) and potassium (K^+^) contents and K^+^/Na^+^ ratio. **(B)** Calcium (Ca^2+^), magnesium (Mg^2+^), phosphorus (P) and sulphur (S) contents. Data are presented as means ± SD (n = 5). Different letters indicate significant differences according to Tukey’s multiple comparison test (P < 0.05).

Analysis of the main macronutrients in leaves revealed genotype-dependent responses to salinity (**Figure 4B**). Ca^2+^ concentrations decreased significantly under salinity in PSK, TI, and PU, while no significant changes were observed in PR and VK. Mg^2+^ concentrations decreased significantly in VK, TI, and PU, but remained unchanged in PR and PSK. Total P concentrations increased significantly in response to salinity in PSK, VK, and TI, while no significant changes were observed in PR and PU. In contrast, total S concentrations decreased significantly in PR, PSK, and VK, but remained unchanged in TI and PU.

The effect of salinity on the main micronutrients in leaves was also analyzed (**Figure 5A**). Fe^2+^ concentrations were not significantly affected by salinity in any of the genotypes. Mn^2+^ concentrations increased significantly in PR, PSK, and TI, decreased significantly in VK, and remained unchanged in PU. Zn^2+^ concentrations increased significantly in PSK and TI, decreased significantly in VK, and remained unchanged in PR and PU. Cu^2+^ concentrations were not significantly affected by salinity in any of the genotypes except PR, where a significant decrease was observed.

**Figure 5:**
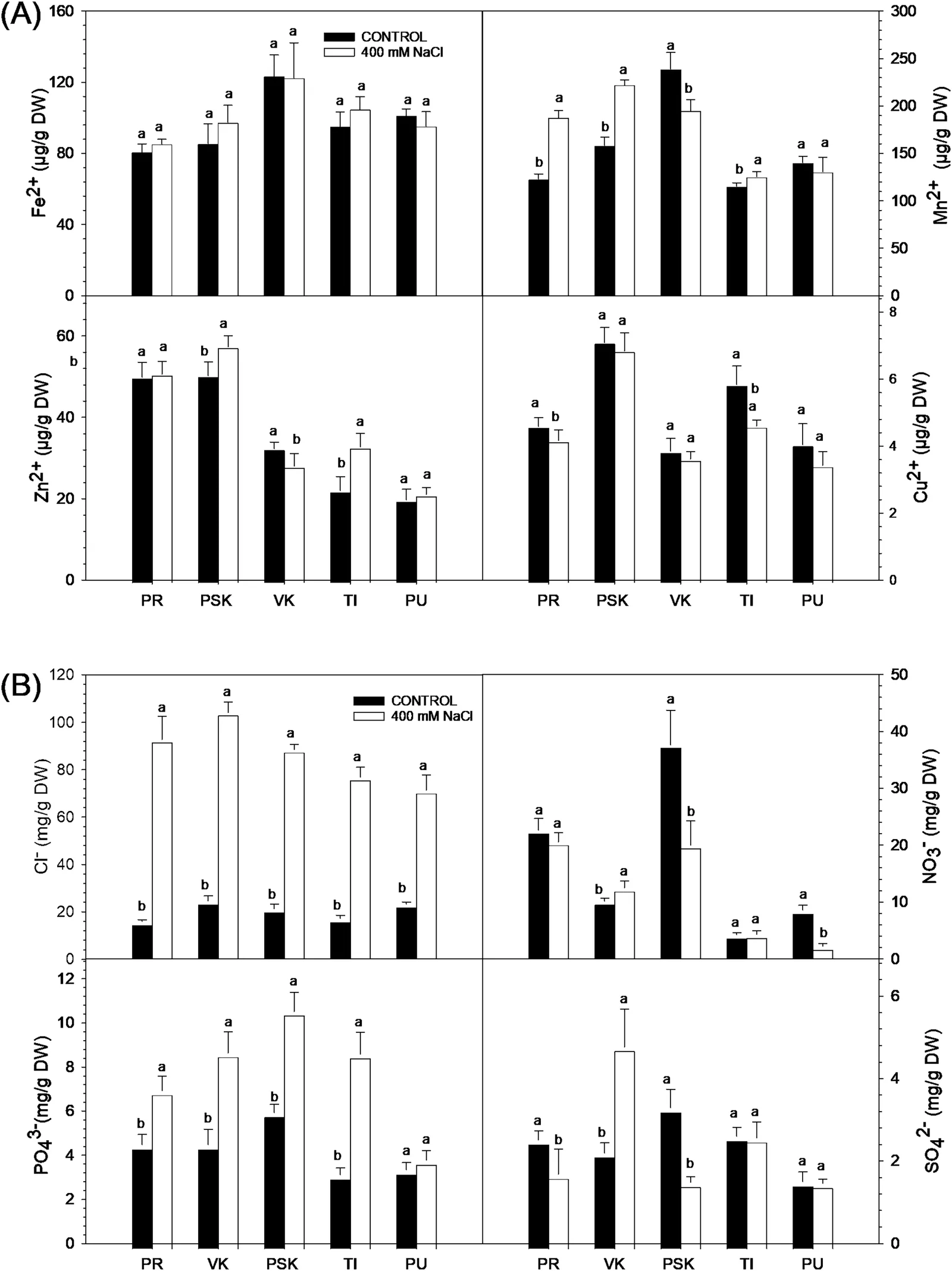
Effect of salinity on mineral composition of leaves from five quinoa genotypes grown under control and 400 mM NaCl conditions. **(A)** Iron (Fe^2+^), manganese (Mn^2+^), zinc (Zn^2+^) and copper (Cu^2+^) contents. **(B)** Chloride (Cl^−^), nitrate (NO_3_^−^), phosphate (PO_4_^3−^) and sulphate (SO_4_^2−^) contents. Data are presented as mean ± SD (n = 5). Different letters indicate significant differences among treatments according to Tukey’s multiple comparison test (P < 0.05).

The effect of salinity on the concentrations of the main anions in leaves was also analyzed (**Figure 5B**). Chloride concentrations increased markedly under salinity in all genotypes. Nitrate concentrations showed a genotype-dependent response to salinity, decreasing significantly in PSK and PU, increasing significantly in VK, and remaining unchanged in PR and TI. Phosphate concentrations showed a more consistent response, increasing significantly in all genotypes except PU, where no significant change was observed. Sulphate concentrations increased significantly only in VK, decreased significantly in PR and PSK, and remained unchanged in TI and PU.

The effect of salinity on total nitrogen and carbon contents, as well as the C/N ratio, was also analyzed in leaves (**Figure 6**). Total nitrogen content decreased significantly under salinity in all genotypes except PR and VK, where no significant changes were observed. Total carbon content decreased significantly in PR, PSK, and TI, increased significantly in VK, and remained unchanged in PU. Finally, the C/N ratio increased significantly in PSK and PU, while no significant changes were observed in the other genotypes.

**Figure 6:**
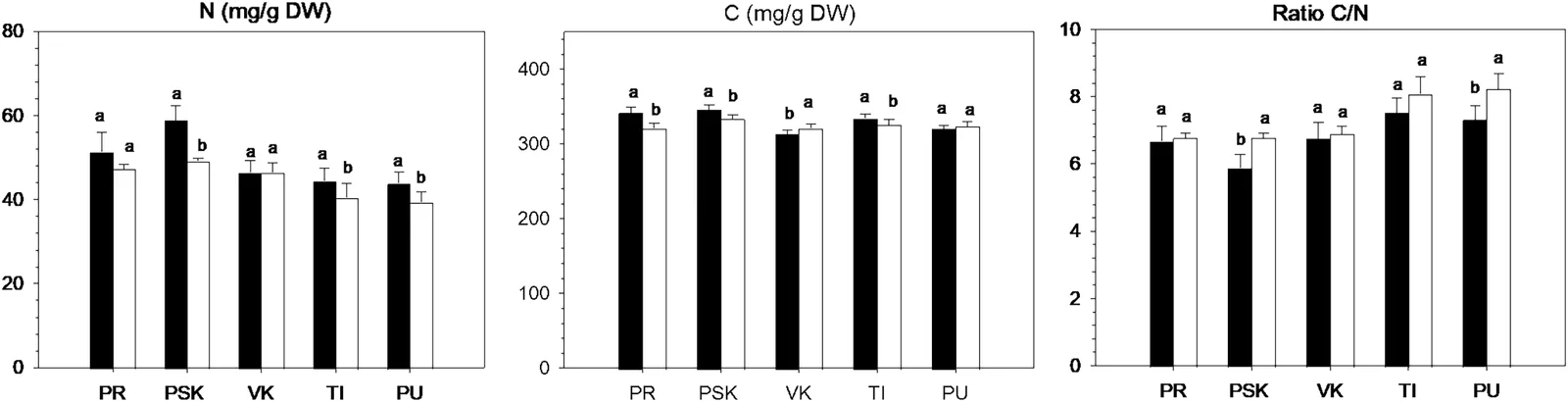
Effect of salinity on Carbon (C), Nitrogen (N) and the ratio C/N in leaves of five quinoa genotypes grown in 400 mM of NaCl. Data are shown in mean ± SD. Different letters indicate the presence of significant differences (P < 0.05) among treatments according to Tukey’s test (n = 5).

Consistently, the genotype × treatment interaction was significant for all ionomic and elemental parameters analyzed in leaves, encompassing cations, anions, and carbon and nitrogen contents (**Supplementary Tables S8–S12**).

### 3.4 Nitrogen allocation to glycinebetaine under salinity

The proportion of total nitrogen allocated to glycinebetaine (%N(GB)) increased significantly under salt stress in all quinoa genotypes (**Table 3**). Under control conditions, glycinebetaine accounted for between 5.46% (PSK) and 8.73% (PU) of total nitrogen, whereas salinity increased this proportion to between 9.70% (PR) and 17.80% (PU). The largest increases were observed in PU and TI, in which glycinebetaine represented 17.80% and 15.03% of total nitrogen, respectively, under 400 mM NaCl. In contrast, the proportion of nitrogen present as nitrate (N% (NO_3_^−^)) showed a genotype-dependent response to salinity. Significant decreases were observed in PSK (14.60 to 9.22%) and PU (4.54 to 1.30%), whereas no significant changes were detected in PR, VK or TI.

**Table 3:** Effect of salinity on the allocation of total nitrogen to glycinebetaine (GB), nitrate (NO_3_^-^) and other nitrogen-containing compounds in five quinoa genotypes.

	Treatment	% N (GB)	% N (NO_3_^-^)	%Nt-%(Ngb+NO_3_)
PR	C	5,54 b	10 a	84,45 a
	400 mM NaCl	9,70 a	9,8 a	80,50 a
VK	C	7,25 b	5,03 a	87,72 a
	400 mM NaCl	12,69 a	6,26 a	81,05 b
PSK	C	5,46 b	14,60 a	79,94 a
	400 mM NaCl	11,68 a	9,22 b	79,10 a
TI	C	7,29 b	2,17 a	90,54 a
	400 mM NaCl	15,03 a	2,40 a	82,57 b
PU	C	8,73 b	4,54 a	86,73 a
	400 mM NaCl	17,80 a	1,30 b	80,90 b

Plants were grown under control conditions (C) or subjected to 400 mM NaCl. %N(GB) and %N(NO_3_^−^) represent the proportions of total nitrogen allocated to glycinebetaine and nitrate, respectively, whereas %Nt − [%N(GB) + %N(NO_3_^−^)] represents the proportion of total nitrogen allocated to compounds other than glycinebetaine and nitrate. Data are presented as mean ± SD (n = 5). Different letters indicate significant differences among treatments according to Tukey’s multiple comparison test (P < 0.05).

The proportion of nitrogen remaining in compounds other than glycinebetaine and nitrate [%Nt − (%N(GB) + %N(NO_3_^−^))] remained remarkably stable in PR and PSK but decreased significantly in VK, TI and PU under salt stress. This reduction resulted primarily from the increased allocation of nitrogen to glycinebetaine, indicating a redistribution of nitrogen metabolism towards the synthesis of this compatible solute. Two-way ANOVA revealed a significant genotype × treatment interaction for all nitrogen distribution parameters, indicating that the effect of salinity on nitrogen allocation differed among genotypes (**Supplementary Table S13**).

### 3.5 Leaf and seed metabolite contents are altered under high-salinity conditions

Primary metabolism encompasses reactions and pathways that are essential for plant growth and development. Using ^1^H-NMR, it is possible to identify and quantify a substantial number of metabolites involved in primary metabolism, including carbohydrates, amino acids and organic acids (Olmos et al., 2024). To evaluate the metabolic response of quinoa leaves and seeds to salinity, we analyzed the effects of 400 mM NaCl.

#### 3.5.1 Seed metabolome

^1^H-NMR analysis of seeds allowed the identification and quantification of 26 metabolites (**Table 4**). The two most abundant metabolites were sucrose and glycinebetaine, both under control and saline conditions. However, the glycinebetaine content was considerably lower than in the leaf. Glutamine was the most abundant amino acid, although its response to salinity was genotype-dependent, increasing in PR and TI, decreasing in PSK, and remaining unchanged in PU and VK. Proline content also showed a genotype-dependent response to salinity, increasing significantly in TI and PU while remaining unchanged in the other genotypes. Regarding carbohydrates, glucose was the second most abundant after sucrose. Under salinity, glucose concentration decreased in PSK, TI, and PU, while it remained unchanged in PR and VK. Sucrose levels remained unchanged in PU and VK, increased in PSK and TI, and decreased in PR. As observed in leaves, malic and citric acids were the most abundant organic acids in seeds. Under salinity, citric acid levels decreased in PR, PSK, and PU, while they remained unchanged in VK and TI. Malic acid levels decreased in PSK and PU and remained unchanged in the other genotypes.

**Table 4:** Metabolite contents (amino acids, carbohydrates, organic acids and other metabolites) in seeds of five quinoa genotypes grown under control (C) and 400 mM NaCl (T) conditions.

mg/g DW	PR		PSK		VK		TI		PU	
	C	T	C	T	C	T	C	T	C	T
Amino acids										
GABA	0.126 a	0.106 b	0.045 b	0.064 a	0.039 b	0.056 a	0.050 b	0.062 a	0.053 a	0.053 a
Ala	0.375 a	0.285 b	0.110 b	0.238 a	0.352 a	0.287 b	0.228 b	0.321 a	0.238 a	0.269 a
Asn	0.116 b	0.153 a	ND	ND	ND	ND	ND	ND	ND	ND
Asp	0.744 a	0.393 b	0.545 a	0.361 b	0.313 a	0.335 a	0.292 a	0.237 b	0.309 a	0.326 a
Glu	1.139 b	1.302 a	0.550 a	0.485 b	1.022 a	1.024 a	0.994 b	1.194 a	1.022 a	1.024 a
Gln	0.392 b	0.708 a	0.098 a	0.051b	0.193 a	0.233a	0.185 a	0.183 a	0.418 a	0.306 b
Ile	0.065 a	0.037 b	0.018 b	0.026 a	0.042 b	0.053 a	0.083 a	0.051 b	0.086 b	0.107 a
Leu	0.052 a	0.037 b	0.013 b	0.020 a	0.037 a	0.035 a	0.068 b	0.145 a	0.140 a	0.060 b
Phe	0.056 a	0.064 a	0.040 a	0.036 a	0.028 b	0.037 a	0.046 b	0.057 a	0.040 a	0.035 a
Pro	0.252 a	0.243 a	0.179 a	0.190 a	0.161 a	0.214 a	0.177 b	0.324 a	0.182 b	0.384 a
Thr	0.057 b	0.104 a	0.026 b	0.130 a	0.055 a	0.055 a	0.063 a	0.066 a	0.072 a	0.067 a
Tyr	0.057 b	0.076 a	0.099 a	0.044 b	0.059 b	0.071 a	0.043 b	0.074 a	0.050 b	0.070 a
Val	0.140 a	0.143 a	0.046 a	0.047 a	0.080 a	0.089 a	0.058 b	0.128 a	0.085 a	0.085 a
Carbohydrate										
Fruc	0.766 a	0.508 b	0.540 a	0.575a	1.160 a	1.077 a	0.752 b	1.241 a	1.093 a	1.292 a
Glc	4.334 a	3.864 a	2.550 a	2.315b	2.720 a	3.061 a	5.092 a	2.715 b	5.548 a	3.803 b
MIto	0.499 a	0.573 a	0.685 b	0.771a	1.011 a	1.206 a	1.182 a	1.044 a	1.486 a	0.853 b
Suc	23.93 a	19.62 b	29.65 b	33.21 a	30.75 a	30.99 a	28.78 b	32.25 a	31.92 a	32.45 a
Organic acids										
Cit	3.521 a	1.842 b	1.344 a	1.169 b	1.018 a	1.007 a	1.913 a	1.788 a	1.562 a	1.267b
Form	0.045 a	0.046 a	0.048 a	0.037 b	0.049 a	0.053 a	0.048 a	0.046 a	0.058 a	0.055 a
Fum	0.018 b	0.021 a	0.019 a	0.011 b	0.007 a	0.006 a	0.011 a	0.008 b	0.012 a	0.011 a
Lac	0.044 b	0.070 a	0.046 a	0.020 b	0.035 b	0.045 a	0.045 a	0.052 a	0.049 a	0.048 a
Mal	0.613 a	0.689 a	1.611 a	1.373 b	0.526 a	0.560 a	0.380 a	0.440 a	0.750 a	0.283b
Succ	0.095 a	0.075 b	0.105 a	0.073 b	0.092 a	0.068 b	0.105 a	0.096 a	0.128 a	0.116 a
Others										
Bet	6.729 b	13.63 a	10.46 b	19.16 a	5.271 b	8.603 a	6.762 b	10.80 a	6.023 b	9.344 a
Cho	0.426 a	0.335 b	0.367 b	0.434 a	0.420 a	0.433 a	0.355 a	0.404 a	0.373 a	0.372 a
Tri	0.108 a	0.061 b	0.113 a	0.104 b	0.091 a	0.089 a	0.079 b	0.092 a	0.066 a	0.077 a

Data are presented as mean ± SD (n = 5). Different letters indicate significant differences among treatments according to Tukey’s multiple comparison test (P < 0.05).

To determine whether high salinity (400 mM NaCl) altered primary metabolism in quinoa seeds, metabolomic profiling was performed by ^1^H-NMR followed by multivariate analysis using PLS-DA. The PLS-DA score plot showed a clear separation among samples according to genotype and salt treatment, revealing metabolic differentiation among the experimental groups (**Figure 7A**).

**Figure 7:**
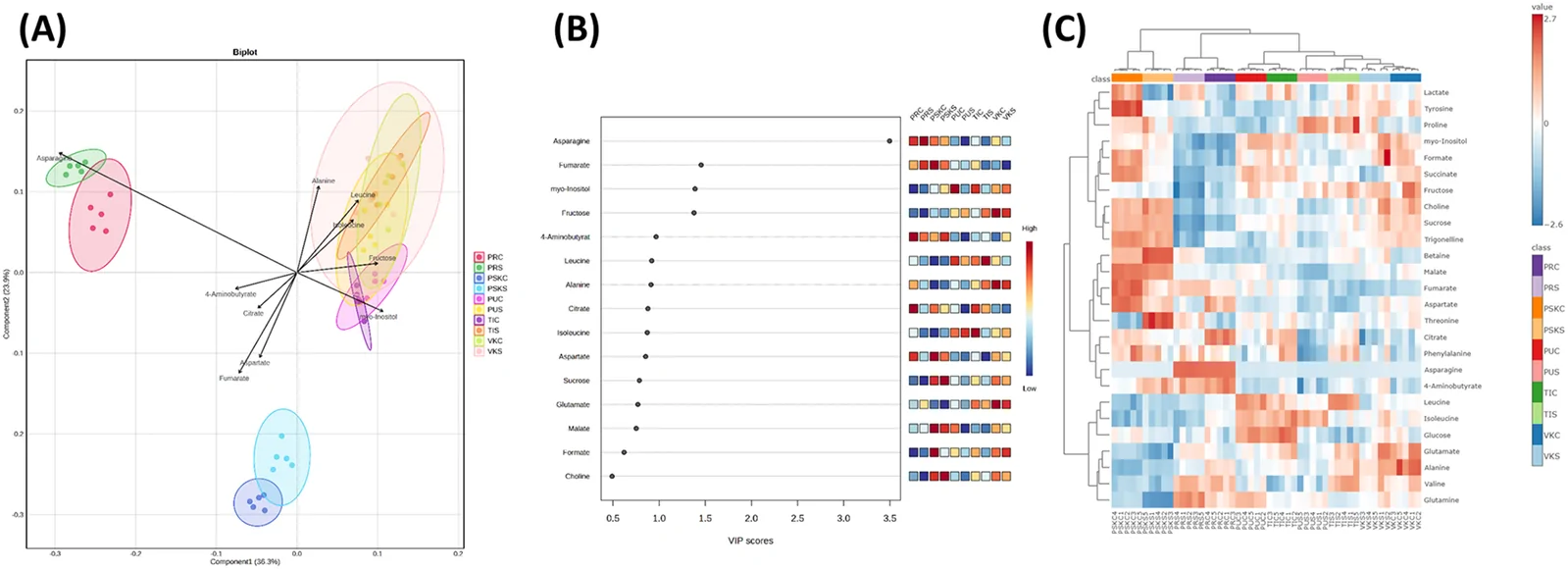
Multivariate analysis of the primary metabolite profiles of seeds from five quinoa genotypes grown under control conditions **(C)** or subjected to 400 mM NaCl (S). **(A)** PLS-DA biplot showing sample distribution and the contribution of individual metabolites to sample discrimination. **(B)** Variable importance in projection (VIP) scores of the metabolites contributing most to sample discrimination; the colored boxes indicate the relative abundance of each metabolite across the experimental groups. **(C)** Hierarchical clustering heatmap of metabolite profiles across genotypes and treatments.

The first two PLS-DA components explained 60.2% of total variance, with component 1 accounting for 36.3% and component 2 for 23.9%. Samples clustered into distinct groups according to genotype and treatment, suggesting strong metabolic differentiation among genotypes. Andean genotypes and European photoperiod-neutral genotypes exhibited different clustering patterns. In particular, PSKC formed an independent cluster separated along negative values of component 1, whereas PRS was clearly differentiated along component 2. By contrast, greater overlap between control and salt-treated samples was observed for the European genotypes examined. The PLS-DA loading plot identified several metabolites contributing to sample discrimination. Positive loadings on component 1 were mainly associated with alanine, leucine, isoleucine, fructose and myo-inositol, whereas negative loadings were related to asparagine, fumarate, aspartate and citrate. These results indicate that metabolites associated with amino acid and carbon metabolism contributed substantially to the discrimination among experimental groups.

Variable Importance in Projection (VIP) analysis identified the metabolites contributing most strongly to sample discrimination (**Figure 7B**). Asparagine exhibited the highest VIP score (~3.4), indicating that this amino acid was the major contributor to the discrimination among experimental groups. Other relevant metabolites included fumarate, myo-inositol, fructose and 4-aminobutyrate (GABA), all showing VIP values >1.5. Additional metabolites contributing to separation included leucine, alanine, citrate, isoleucine and aspartate. The relative abundance patterns of VIP metabolites revealed contrasting responses among genotypes. Salinity was associated with increased levels of myo-inositol, fructose, alanine and branched-chain amino acids (leucine and isoleucine) in several genotypes, whereas asparagine, fumarate and citrate tended to decrease under saline conditions. However, these responses varied markedly among genotypes, indicating genotype-dependent differences in metabolic responses to severe salinity.

Hierarchical clustering analysis combined with heatmap visualization confirmed the genotype-dependent metabolic responses (**Figure 7C**). Samples grouped primarily according to genotype rather than treatment, indicating a strong genetic influence on seed metabolite composition. Nevertheless, differences between control and salt-treated samples were also observed within individual genotypes. The heatmap showed two major clusters of metabolites. One cluster comprised metabolites associated with nitrogen metabolism and osmotic adjustment, including proline, myo-inositol, betaine, trigonelline and amino acids, whereas another cluster included compounds involved in central carbon metabolism, such as organic acids (fumarate, malate, citrate and succinate) and soluble sugars. Salt-treated samples generally showed higher relative abundances of several metabolites associated with osmoprotection and amino acid metabolism, whereas several TCA-cycle intermediates showed lower relative abundances.

#### 3.5.2 Leaf metabolome

^1^H-NMR analysis of leaves allowed the identification and quantification of 27 metabolites (**Table 5**). As observed in the leaf metabolome, sucrose and glycinebetaine were the two most abundant metabolites under both control and saline conditions. The high glycinebetaine levels reached under salt treatment, which ranged between 4% and 6% of dry matter, are particularly noteworthy. The most abundant amino acids in leaves were Asp, Glu and Gln. Their concentrations tended to decrease in response to salt treatment (**Table 5**). However, proline contents increased significantly in all genotypes except PSK, in which no significant changes were observed. Carbohydrate metabolism also differed among genotypes. Fructose increased significantly in PSK and TI but decreased in PR and PU, whereas glucose accumulated only in PSK and TI but decreased in VK. Sucrose remained relatively stable in all genotypes. Organic acids exhibited contrasting responses to salinity. Citrate increased in PR and VK but decreased markedly in PSK and PU. Malate and succinate generally declined under salt treatment, although malate remained unchanged in PR and VK. Similarly, fumarate decreased in PSK and PU but remained relatively stable in the other genotypes.

**Table 5:** Metabolite contents (amino acids, carbohydrates, organic acids and other metabolites) in leaves of five quinoa genotypes grown under control (C) and 400 mM NaCl (T) conditions.

mg/g DW	PR		PSK		VK		TI		PU	
	C	T	C	T	C	T	C	T	C	T
Amino acids										
GABA	0.081 a	0.039 b	0.206 b	0.676 a	0.079 a	0.078 a	0.160 a	0.241 a	0.092 a	0.050 b
Ala	0.472 a	0.598 a	0.546 a	0.456 a	0.621 a	0.455 b	0.616 a	0.484 a	0.571 a	0.361 b
Asn	0.162 a	0.117 a	0.230 a	0.104 b	ND	ND	ND	ND	0.0707	ND
Asp	1.013 b	1.414 a	1.167 a	0.701 b	1.952 a	1.187 b	2.034 a	0.818 b	2.033 a	0.528 b
Glu	4.060 a	3.058 b	3.386 a	1.858 a	2.916 a	1.925 b	3.671 a	1.676 b	3.163 a	1.427 b
Gln	1.062 a	0.856 b	ND	0.190 a	1.055 a	0.805 a	1.236 a	0.473 b	1.215 a	0.573 b
Gly	0.575 a	0.552 a	0.324 a	0.230 b	0.559 a	0.510 a	0.762 a	0.432 b	0.837 a	0.660 b
Ile	0.216 a	0.227 a	0.056 b	0.159 a	0.059 b	0.074 a	0.113 a	0.090 a	0.122 a	0.071 b
Leu	0.194 a	0.066 b	0.236 a	0.190 a	0.121 a	0.049 b	0.058 b	0.163 a	0.093 a	0.125 a
Phe	0.216 a	0.044 b	0.189 a	0.109 b	0.053 a	0.036 a	ND	0.071	0.082	ND
Pro	0.297 b	0.582 a	0.344 a	0.379 a	0.215 b	0.446 a	0.149 b	0.358 a	0.223 b	0.442 a
Thr	0.288 a	0.184 b	0.536 a	0.387 b	0.207 a	0.165 b	0.221 a	0.181 b	0.234 a	0.121 b
Tyr	0.245 a	0.085 b	0.109 a	0.072 a	0.112 a	0.051 b	0.094 b	0.264 a	0.120 a	0.101 a
Val	0.257 a	0.076 b	0.191 a	0.179 a	0.074 a	0.060 a	0.093 a	0.124 a	0.091 a	0.098 a
Carbohydrates										
Fruc	2.187 a	1.054 b	1.508 b	1.870 a	0.921 a	0.954 a	0.935 b	2.201 a	2.142 a	1.432 b
Glc	2.353 a	2.249 a	1.874 b	2.692 a	2.171 a	1.351b	2.002 b	4.698 a	1.721 a	1.809 a
MaIt	0.462 a	0.417 a	0.539 b	0.708 a	0.221 a	0.189 a	0.136 a	0.148 a	0.285 a	0.145 b
Suc	13.25 a	12.46 a	6.398 a	6.333 a	14.52 a	12.70 a	13.89 a	14.71 a	19.02 a	17.22 a
Organic acids										
Cit	0.729 b	1.089 a	4.308 a	2.017 b	0.382 b	0.584 a	3.720 a	4.585 a	2.298 a	1.145 b
Form	0.086 a	0.083 a	0.070 a	0.066 a	0.053 b	0.069 a	0.085 a	0.072 a	0.058 b	0.079 a
Fum	0.023 a	0.025 a	0.041 a	0.023 b	ND	0.014	0.031 a	0.026 a	0.024 a	0.017 b
Mal	1.887 a	2.121 a	5.276 a	3.011 b	0.981 a	0.956 a	3.793 a	2.782 b	3.602 a	2.830 b
Succ	0.199 a	0.140 b	0.205 a	0.106 b	0.037 a	0.013 b	0.296 a	0.144 b	0.198 a	0.116 b
Others										
Bet	23.68 b	40.08 a	28.20 b	48.80 a	26.90 b	48.06 a	27.08 b	49.60 a	31.92 b	58.59 a
Cho	0.674 a	0.607 a	0.875 a	0.835 a	0.464 a	0.467 a	0.278 b	0.660 a	0.469 a	0.495 a
Ea	0.082 a	0.066 b	0.130 a	0.101 a	ND	ND	ND	ND	ND	ND
Tri	0.478 a	0.381 b	0.495 a	0.435 a	0.351 a	0.360 a	0.396 b	0.581 a	0.379 b	0.492 a

Data are presented as mean ± SD (n = 5). Different letters indicate significant differences among treatments according to Tukey’s multiple comparison test (P < 0.05).

Metabolic profiling by ^1^H-NMR identified clear differences among the five quinoa genotypes and between control and salt-treated plants. The PLS-DA score plot showed a distinct separation of the samples, with Component 1 explaining 44.2% of the total variance and Component 2 explaining 10.9% (**Figure 8A**). The first component contributed mainly to the discrimination among experimental groups, whereas the second component captured additional metabolic variation among samples. Among the analyzed genotypes, PRC and PRS clustered on the negative side of Component 1, whereas VKC, VKS and TitiS were mainly located on the positive side. The salt-treated samples generally showed a displacement relative to their corresponding controls, although the magnitude of the response varied among genotypes. Among the experimental groups, PUC showed the greatest dispersion in the PLS-DA score plot. The loading vectors indicated that several metabolites contributed to sample discrimination. Positive values of component 1 were mainly associated with higher levels of citrate, malate, fumarate, glutamine and glycinebetaine, whereas negative values were associated with phenylalanine, asparagine, ethanolamine and maltose. 4-Aminobutyrate (GABA) contributed predominantly to the separation along Component 2.

**Figure 8:**
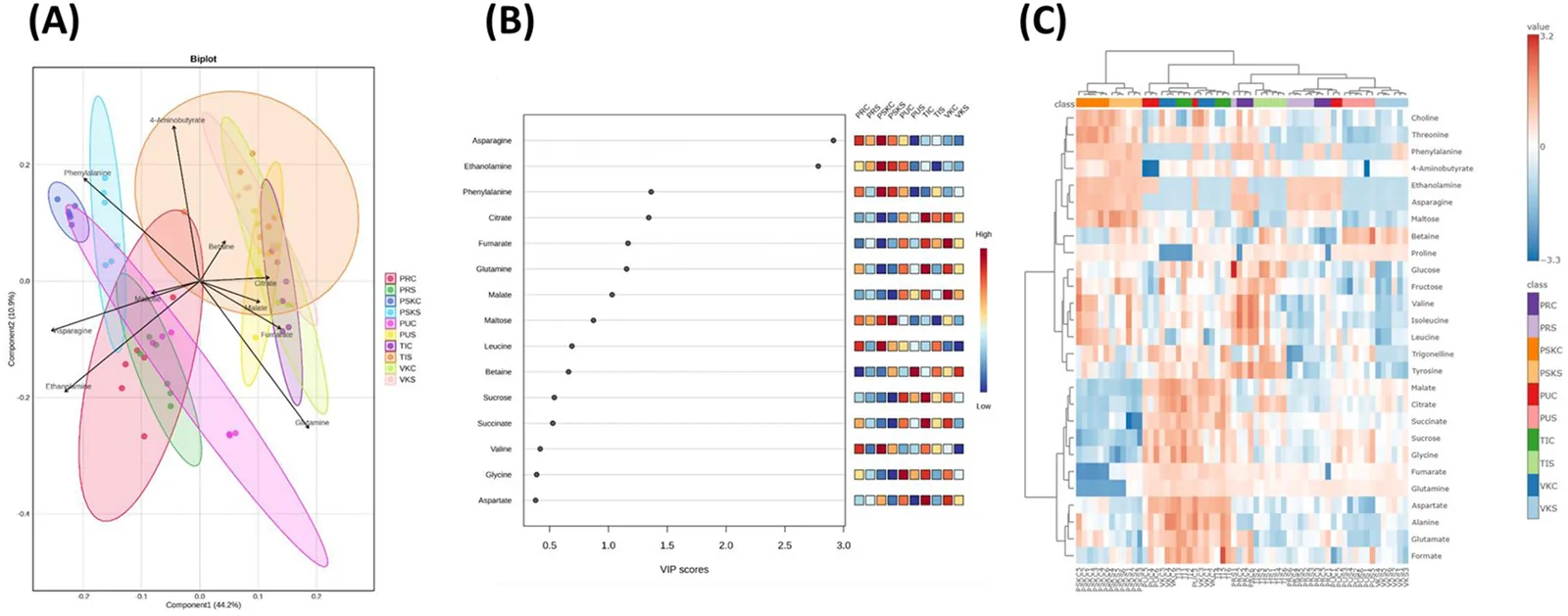
Multivariate analysis of the primary metabolite profiles of leaves from five quinoa genotypes grown under control conditions **(C)** or subjected to 400 mM NaCl (S). **(A)** PLS-DA biplot showing sample distribution and the contribution of individual metabolites to sample discrimination. **(B)** Variable importance in projection (VIP) scores of the metabolites contributing most to sample discrimination; the colored boxes indicate the relative abundance of each metabolite across the experimental groups. **(C)** Hierarchical clustering heatmap of metabolite profiles across genotypes and treatments.

The VIP analysis identified the metabolites with the highest discriminatory power (**Figure 8B**). Asparagine and ethanolamine showed the highest VIP scores (approximately 2.8-3.0), followed by phenylalanine, citrate, fumarate, glutamine and malate. Metabolites such as maltose, leucine, betaine, sucrose, succinate, valine, glycine and aspartate also contributed to the discrimination among samples, although with lower VIP values. The heatmap confirmed the clustering observed in the PLS-DA (**Figure 8C**). Samples grouped according to both genotype and treatment, while metabolites exhibited distinct accumulation patterns. A cluster including choline, threonine, phenylalanine, 4-aminobutyrate, ethanolamine and asparagine accumulated preferentially in PRC and PRS samples but generally decreased in the remaining genotypes, particularly under salt treatment. In contrast, another cluster containing citrate, malate, succinate, fumarate, glutamine, glycine, aspartate and alanine showed higher relative abundance in salt-treated samples of several genotypes, indicating that metabolites associated with carbon and nitrogen metabolism contributed to the differences observed among experimental groups. Sugars such as glucose, fructose and sucrose displayed intermediate patterns, with genotype-dependent variation in their accumulation.

The PLS-DA models showed a high goodness of fit and predictive ability for both leaf (R² = 0.913, Q² = 0.835) and seed (R² = 0.962, Q² = 0.925) metabolite profiles (**Supplementary Tables S14, 15**). Permutation testing supported the statistical significance of both models, with none of the 1,000 permuted models reaching the observed test statistic (P < 0.001; 0/1000) (**Supplementary Figure S2**). As the PLS-DA discrimination reflects variation associated with both genotype and salinity treatment, the effects of these factors and their interaction were further assessed by two-way ANOVA. A significant genotype × treatment interaction was detected for all metabolites analyzed in both leaves and seeds, indicating that their responses to severe salinity differed among genotypes (**Supplementary Tables S16–23**).

## 4 Discussion

### 4.1 High salinity induced a significant reduction in plant biomass and grain yield in quinoa

A plant is considered halophytic when it is able to complete its life cycle in environments where the salt concentration is 200 mM or higher (Flowers and Colmer, 2008). Quinoa is considered a facultative halophyte, as it does not require a saline medium for growth but is able to grow and complete its vegetative cycle at concentrations as high as 400 mM NaCl (Adolf et al., 2012; Roman et al., 2020). Quinoa may benefit from moderate salinity, with previous studies reporting greater growth and biomass accumulation at approximately 100–200 mM NaCl than under non-saline conditions (Hariadi et al., 2011). Although vegetative growth was markedly reduced in all genotypes, with reductions of approximately 36-60%, it is worth noting that grain yield per plant was less affected, showing reductions of approximately 18-35%. This led to an increase in harvest index in most genotypes. In a previous study, Adolf et al. (2012) reported similar results for the PSK, Puno and Titicaca genotypes, with biomass reductions of approximately 45%. However, the PR genotype was more sensitive in our study. This may be because PR showed a more prolonged vegetative growth phase, as it was not grown under optimal photoperiod conditions. Similarly, the andean short-photoperiod genotypes showed lower grain yield under both control and saline conditions. Therefore, all European genotypes showed high grain yield per plant under both control and saline conditions, with values approximately twice those of the andean genotypes.

However, our results contrast with those reported by Roman et al. (2020), in which European genotypes (Jessie, Pasto and Riobamba) grown under 400 mM NaCl showed a drastic reduction in growth and seed production, of approximately 88%, although they were still able to complete their life cycle. Nevertheless, the harvest index of Riobamba and Jessie was not significantly affected, whereas that of Pasto was strongly reduced. In addition, Toderich et al. (2020) observed that a salt-tolerant cultivar, Q5, grown in soil with high sodium (16.2 mM/100 g soil) and sulphate (14.5 mM/100 g soil) levels showed a 30% reduction in plant dry weight and a 37% reduction in seed yield per plant. Therefore, our results suggest that although andean short-photoperiod genotypes such as PR and PSK are able to complete their life cycle and grow under high-salinity conditions in long-photoperiod regions, they are less suitable than European genotypes for cultivation under the climatic conditions of Mediterranean countries. However, the available data do not allow us to determine whether the poorer growth performance observed in the Andean genotypes can be attributed to differences in photoperiod sensitivity.

### 4.2 Ion homeostasis is altered under high-salinity stress in quinoa plants

The mineral composition of both leaves and seeds was markedly affected by severe salinity, although the magnitude and direction of these changes were strongly dependent on genotype and organ. Reductions in Ca^2+^ and Mg^2+^, particularly in leaves, may result from interference of Na^+^ with the uptake and transport of divalent cations, as previously reported in quinoa under saline conditions (Rezzouk et al., 2022). In contrast, P concentrations were maintained or increased in most genotypes, suggesting that P acquisition and redistribution remained relatively well preserved despite exposure to 400 mM NaCl.

Karyotis et al. (2003) reported that seeds from plants grown in saline-sodic soils in Greece contained lower concentrations of calcium, magnesium, zinc and manganese than those produced under non-saline conditions. In contrast, Toderich et al. (2020) observed increased concentrations of calcium, iron and zinc in seeds from salt-treated plants. Na^+^ and Cl^−^ accumulated in all genotypes, consistent with the halophytic behavior of quinoa. Previous studies have shown that, in this species, inorganic ions, particularly Na^+^, K^+^ and Cl^−^, can contribute to osmotic adjustment, reducing the metabolic cost associated with the synthesis of organic osmolytes (Hariadi et al., 2011). Nevertheless, substantial genotypic differences were observed, particularly in seeds, where PR accumulated more than twice as much Na^+^as the other genotypes and showed an almost sevenfold increase in Cl^−^. Previous studies have similarly reported considerable genotypic variation in ion uptake, exclusion and translocation in quinoa under high salinity (Adolf et al., 2012; Roman et al., 2020). The accumulation of Na^+^ and Cl^−^ in seeds is particularly relevant because excessive deposition of these ions in reproductive tissues may compromise seed development and germination. Previous studies have demonstrated that quinoa can restrict the movement of potentially toxic ions into metabolically active seed tissues. Using scanning electron microscopy coupled with energy-dispersive X-ray (SEM-EDX) microanalysis, both Koyro and Eisa (2008) and Eisa et al. (2017) demonstrated that Na^+^ accumulated predominantly in the seed pericarp. Koyro and Eisa (2008) reported that more than 90% of Na^+^ and Cl^−^ was retained in the pericarp under high salinity, while Eisa et al. (2017) detected only trace amounts of Na^+^ in the perisperm and embryo.

### 4.3 Effect of high salinity in primary metabolome in quinoa leaves and seeds

One of the most common responses of halophytic species to salinity is the accumulation of inorganic ions in the vacuole, and compounds with osmotic or osmoprotective functions in the cytoplasm, such as soluble sugars and polyols (Slama et al., 2015). In quinoa leaves, sucrose was the most abundant carbohydrate; however, its concentration was not significantly affected by salt treatment, whereas glucose and fructose concentrations either remained unchanged or showed genotype-dependent changes. However, a previous study conducted on quinoa plants grown under controlled conditions and subjected to short-term salinity (7 and 14 days) showed a decrease in sucrose concentration accompanied by increased glucose and fructose concentrations (Olmos et al., 2024). However, sugar accumulation does not appear to be a universal response of quinoa to salinity. Kiani-Pouya et al. (2017) reported substantial decreases in glucose and sucrose in salt-treated quinoa leaves, whereas inositol increased. Thus, the contrasting sugar responses observed here probably reflect differences in carbon utilization and partitioning among genotypes rather than a common osmotic response.

Amino acid metabolism was also strongly affected. Aspartate, glutamate and glutamine were among the most abundant amino acids in leaves and generally tended to decrease under salinity. Kiani-Pouya et al. (2017) showed that salinity induced significant changes in the amino acid profile of quinoa. Proline concentration increased sharply, reaching levels approximately 16-fold higher than those of control plants, whereas aspartate concentration decreased considerably. In our study, although proline concentration increased in most genotypes, the increase was more moderate, reaching approximately twice the control values. The accumulation of proline in response to salinity and other abiotic stresses has been widely documented (Mansour and Ali, 2017; Iqbal et al., 2019). In a previous study using selected tobacco cells exposed to high salt concentrations, we observed a marked accumulation of proline, reaching exceptionally high levels (de la Garma et al., 2015). Proline has been proposed to contribute to osmotic adjustment and to act as an osmoprotectant, helping to stabilize cellular structures and maintain protein integrity under stress conditions (Mansour and Ali, 2017).

One of the clearest responses involved organic acids. Malate, succinate and fumarate generally decreased in leaves, although the response of citrate was strongly genotype-dependent. A similar depletion of organic acids has previously been observed in salt-treated quinoa, where citrate, fumarate and malate decreased markedly (Kiani-Pouya et al., 2017). Olmos et al. (2024) also reported that short-term salt stress resulted in decreased malate and succinate concentrations, accompanied by an increase in citrate concentration. The effect of salinity on organic acids, particularly those involved in the TCA cycle, was reflected in a general decrease in their concentrations. These changes may be associated with alterations in TCA cycle activity and respiratory carbon flux, as proposed in previous studies, as well as with increased utilization of carbon skeletons in stress-related metabolic processes (Kumari and Parida, 2018). However, metabolic fluxes were not directly assessed in the present study.

Interestingly, genotype appeared to exert a stronger influence on the seed than on the leaf metabolome. Andean and European genotypes displayed different clustering patterns, with the European genotypes generally showing greater overlap between control and salt-treated seeds. The metabolic composition of quinoa seeds appears to be strongly genotype-dependent. To our knowledge, little information is available on the effect of salinity during plant growth on the metabolome in mature quinoa seeds. Using a ^1^H-NMR-based metabolomic approach, Lalaleo et al. (2020) identified 30 metabolites in seeds from three quinoa ecotypes and showed a clear discrimination among ecotypes based on their metabolic profiles, irrespective of seed treatment. A previous ^1^H NMR-based metabolomic study of quinoa seeds from different geographical origins subjected to drought stress showed that sucrose was the most abundant compound in all genotypes and that its concentration increased markedly in response to stress (Matías et al., 2023).

### 4.4 Glycinebetaine and nitrogen metabolism under salt stress

Evidence regarding the effect of salinity on nitrogen metabolism in quinoa remains scarce. Miranda-Apodaca et al. (2020) observed that high salinity (500 mM NaCl) induced a decrease in both total nitrogen and leaf nitrate, possibly owing to increased inhibition of nitrogen uptake and nitrogen transport to the stem. In general, in the present study, we also observed a trend towards salinity-induced reduction in total leaf nitrogen.

In plants belonging to the families Amaranthaceae, Chenopodiaceae and Malvaceae, all of which include glycinebetaine-accumulating species, glycinebetaine can account for up to 10% of total leaf nitrogen under drought stress conditions in semi-arid mulga forests of Australia (Erskine et al., 1996). Similarly, glycinebetaine represented up to 18% of total nitrogen in quinoa leaves under saline conditions. By assessing the contribution of glycinebetaine and nitrate to total leaf nitrogen, we observed contrasting patterns of nitrogen distribution among the genotypes examined. Under saline conditions, the three European genotypes included in this study showed a greater proportion of nitrogen associated with glycinebetaine and a relatively low contribution of nitrate. The two Andean genotypes showed a comparatively greater contribution of nitrate, although this remained lower than that associated with glycinebetaine. These patterns should be interpreted as specific to the genotypes examined rather than as a general effect of geographical origin.

Glycinebetaine can account for up to 4-6% of total leaf dry weight under salinity. Glycinebetaine synthesis requires a substantial energy supply, and this compound is not catabolized by plants (McNeil et al., 2001; Valenzuela-Soto and Figueroa-Soto, 2019). Proline and glycinebetaine are generally regarded as the principal osmolytes and osmoprotectants in many plant species (Ashraf and Foolad, 2007; Annunziata et al., 2019). However, the substantially lower concentration of proline relative to glycinebetaine in quinoa leaves suggests that proline contributes only marginally to osmotic adjustment and osmoprotection in this species (Olmos et al., 2024). Our results show that salinity markedly altered the distribution of leaf nitrogen, with an increased proportion of total nitrogen associated with glycinebetaine under stress conditions. Together with the exceptionally high glycinebetaine concentrations measured in leaves, this association suggests that glycinebetaine accumulation may represent an important component of the biochemical response of quinoa to severe salinity. However, the present data do not establish a causal role for glycinebetaine in salt tolerance.

Information on the glycinebetaine content of quinoa seeds is very limited. Recently, an untargeted metabolomic analysis of seeds from 471 quinoa cultivars revealed the presence of glycinebetaine in all genotypes (Tabatabaei et al., 2022). However, its concentration could not be quantified, although the data suggested that it was among the most abundant metabolites. According to Ross et al. (2014), the concentration of glycinebetaine in quinoa seeds is approximately 3,930 μg g^-1^, whereas the USDA database estimates it at 6,000 μg g^-1^ (Patterson et al., 2008). Our results showed concentrations comparable to those previously reported, although salt stress caused a significant increase, with values reaching 19 mg g^-1^ DW in PSK. Other stresses, such as drought, have also been shown to significantly increase glycinebetaine levels in quinoa seeds (Matías et al., 2023).

In seeds, glycinebetaine concentrations were much lower than in leaves, approximately 2.5 to 6-fold lower, but salt treatment induced glycinebetaine accumulation in the seeds of all genotypes. Glycinebetaine is synthesized in chloroplasts by the sequential action of choline monooxygenase (CMO) and betaine aldehyde dehydrogenase (BADH) and is subsequently transported to the cytoplasm and other plant organs (Annunziata et al., 2019). It has been proposed that glycinebetaine is translocated from its site of synthesis to active growth zones through phloem transport (Chen and Murata, 2011). However, proteomic analyses of quinoa seeds have revealed the significant presence of CMO (Poza-Viejo et al., 2023), suggesting that glycinebetaine synthesis may occur, at least to some extent, during grain filling. As observed in the present study, the salinity-induced increase in glycinebetaine at the leaf level was accompanied by increased accumulation in seeds. Although germination performance under saline conditions was not evaluated here, this accumulation raises the hypothesis that seed glycinebetaine could contribute to osmotic adjustment during germination, potentially favoring seedling establishment under saline conditions. Similarly, the higher glycinebetaine content of seeds produced under salinity could potentially influence their nutritional or nutraceutical properties. However, these possibilities remain hypothetical, as neither germination performance nor the nutritional and health-related effects associated with seed glycinebetaine accumulation were directly assessed in the present study.

Glycinebetaine plays an important role in human health, both as an osmolyte and as a methyl-group donor. Although it is not classified as an essential nutrient, humans do not synthesize sufficient quantities of glycinebetaine, and therefore dietary intake is required. Glycinebetaine has been shown to act as a methyl donor in the reduction of circulating homocysteine levels, which are considered a biomarker of increased risk of vascular disease (González-Lamuño et al., 2024). Several studies have shown that dietary supplementation with glycinebetaine reduces circulating homocysteine levels (Ashtary-Larky et al., 2022). Recently, a study involving 5,613 Spanish adults participating in the PREDIMED-Plus program over a one-year period investigated the association between dietary choline and glycinebetaine intake and cardiometabolic health. The authors demonstrated that higher glycinebetaine intake was positively associated with improved cardiovascular parameters and renal function (Diez-Ricote et al., 2022). The main sources of glycinebetaine in Western diets are cereal grains, particularly wheat (Ross et al., 2014; Filipcev et al., 2018). Because people with coeliac disease cannot consume gluten-containing products such as wheat-based foods, deficiencies in daily glycinebetaine intake may occur. Therefore, the incorporation of quinoa, a gluten-free species, into the diet could substantially help compensate for this lack of glycinebetaine in coeliac diets. The use of genotypes with high glycinebetaine levels in the seed could therefore represent an interesting trait for selection in future quinoa breeding programs.

Overall, the Andean and European genotypes examined in this study showed both common and contrasting responses to severe salinity. Growth and yield responses were predominantly genotype-dependent rather than consistently associated with geographical origin, as the largest reductions in plant biomass, height growth, and seed yield occurred in different European genotypes, while PR showed the smallest reduction in seed yield. However, given the limited number of genotypes representing each geographical group, the observed differences between Andean and European genotypes cannot be unequivocally attributed to geographical origin and may instead reflect the specific genetic backgrounds of the genotypes examined. Similarly, changes in seed physical traits were largely genotype-dependent, with PR showing the greatest reduction in seed size and seed weight, whereas some European genotypes were comparatively less affected. Nevertheless, the European genotypes consistently exhibited a higher harvest index than the Andean genotypes under both control and saline conditions, indicating a greater relative allocation of biomass to reproductive output. Contrasting patterns of K^+^ partitioning was observed among the genotypes examined. Under salinity, seed K^+^ concentrations increased in the two Andean genotypes included in this study but remained unchanged in the three European genotypes, whereas leaf K^+^ was maintained or increased in the European genotypes but decreased or remained unchanged in the Andean genotypes. Although this contrasting pattern was associated with the geographical groups represented in our study, it cannot be attributed to geographical origin *per se* and may instead reflect the specific genetic backgrounds of the genotypes examined. Other mineral and metabolic responses were largely genotype-specific, highlighting substantial variation within each geographical group. Although salinity increased nitrogen allocation to glycinebetaine in all genotypes, contrasting patterns of soluble nitrogen partitioning were observed among the genotypes examined. The three European genotypes included in this study showed a greater allocation of nitrogen to glycinebetaine, whereas the two Andean genotypes retained a greater contribution of nitrate. However, this pattern should be interpreted as specific to the genotypes examined and cannot be attributed to geographical origin *per se*. Taken together, these findings indicate that the response of quinoa to severe salinity involves common responses, such as enhanced glycinebetaine accumulation, together with genotype-dependent differences in growth, reproductive performance, ion homeostasis, nitrogen distribution, and primary metabolism.

## Data Availability

The raw data supporting the conclusions of this article will be made available by the authors, without undue reservation.
