# Supplementary material for: Severe salinity reshapes the ionomic and metabolic profiles of quinoa leaves and seeds in a genotype-dependent manner
Source: Front Plant Sci. 2026 Oct 19;17:1991117. doi: 10.3389/fpls.2026.1991117 (PMC13725696; doi:10.3389/fpls.2026.1991117)
Supplement: Supplementary file 1 [file Table1.docx]

**SUPPLEMENTARY TABLES**

**Table S1.** Plant DW, seed yield and harvest index (HI). A Two-Way ANOVA followed by a Tukey post-hoc test was performed to analyse the effects of treatment, genotype and their interaction on the parameters measured. Significance levels are indicated as follows: ns, not significant; *P < 0.05; **P < 0.01; and ***P < 0.001 (n = 10).

|  | Plant DW (g) | Seed yield (g plant^-1^) | *Harvested index* (%) |
| --- | --- | --- | --- |
| Treatment (T) | *** | *** | *** |
| Genotype (G) | ns | *** | *** |
| T x G | *** | *** | *** |

**Table S2.** Seed length, 100 seed weight and % of dry weight. A Two-Way ANOVA followed by a Tukey post-hoc was performed to analyse the influence of the treatment, and genotype and their interaction in the different parameter measured in this study. Significance levels: ns = not significant; * P < 0.05; ** P < 0.01; and *** P < 0.001 (n = 100).

|  | Seed length (mm) | 100 seed weight (g) | % Dry weigth |
| --- | --- | --- | --- |
| Treatment (T) | ** | ** | ns |
| Genotype (G) | *** | *** | *** |
| T x G | *** | *** | *** |

**Table S3.** Ionic contents in quinoa seeds (Na^+^ and K^+^ and the ratio Na/K). A Two-Way ANOVA followed by a Tukey post-hoc was performed to analyse the influence of the treatment, and genotype and their interaction in the different parameter measured in this study. Significance levels: ns = not significant; * P < 0.05; ** P < 0.01; and *** P < 0.001 (n = 5).

|  | K^+^ (mg/g DW) | Na^+^ (mg/g DW) | Ratio K/Na |
| --- | --- | --- | --- |
| Treatment (T) | *** | *** | *** |
| Genotype (G) | * | ** | ns |
| T x G | *** | *** | *** |

**Table S4.** Ionic contents in quinoa seeds (Ca^2+^, Mg^2+^, P, and S). A Two-Way ANOVA followed by a Tukey post-hoc was performed to analyse the influence of the treatment, and genotype and their interaction in the different parameter measured in this study. Significance levels: ns = not significant; * P < 0.05; ** P < 0.01; and *** P < 0.001 (n = 5).

|  | Ca^2+^ (mg/g DW) | Mg^2+^ (mg/g DW) | P (mg/g DW) | S (mg/g DW) |
| --- | --- | --- | --- | --- |
| Treatment (T) | ns | * | *** | ns |
| Genotype (G) | *** | ** | *** | *** |
| T x G | *** | *** | *** | *** |

|  | Fe^2+^ (µg/g DW) | Mn^2+^ (µg/g DW) | Zn^2+^ (µg/g DW) | Cu^2+^ (µg/g DW) |
| --- | --- | --- | --- | --- |
| Treatment (T) | ns | ** | ns | ns |
| Genotype (G) | *** | ** | *** | *** |
| T x G | *** | *** | *** | *** |

**Table S5.** Ionic contents in quinoa seeds (Fe^2+^, Mn^2+^, Zn^2+^ and Cu^2+^). A Two-Way ANOVA followed by a Tukey post-hoc was performed to analyse the influence of the treatment, and genotype and their interaction in the different parameter measured in this study. Significance levels: ns = not significant; * P < 0.05; ** P < 0.01; and *** P < 0.001 (n = 5).

**Table S6.** Ionic contents in quinoa seeds (Cl^-^, NO_3_^-^, PO_4_^3-^ and SO_4_^2-^). A Two-Way ANOVA followed by a Tukey post-hoc was performed to analyse the influence of the treatment, and genotype and their interaction in the different parameter measured in this study. Significance levels: ns = not significant; * P < 0.05; ** P < 0.01; and *** P < 0.001 (n = 5).

|  | Cl^-^ (mg/g DW) | NO_3_^-^ (mg/g DW) | PO_4_^3-^ (mg/g DW) | SO_4_^2-^ (mg/g DW) |
| --- | --- | --- | --- | --- |
| Treatment (T) | *** | * | ns | ns |
| Genotype (G) | *** | *** | *** | *** |
| T x G | *** | *** | *** | *** |

**Table S7.** Ionic contents in quinoa seeds (Ct, Nt and the ratio C/N). A Two-Way ANOVA followed by a Tukey post-hoc was performed to analyse the influence of the treatment, and genotype and their interaction in the different parameter measured in this study. Significance levels: ns = not significant; * P < 0.05; ** P < 0.01; and *** P < 0.001 (n = 5).

|  | C total (mg/g DW) | N total (mg/g DW) | Ratio C/N |
| --- | --- | --- | --- |
| Treatment (T) | *** | ns | ns |
| Genotype (G) | *** | *** | *** |
| T x G | *** | *** | *** |

**Table S8.** Ionic contents in quinoa leaves (K^+^ and Na^+^). A Two-Way ANOVA followed by a Tukey post-hoc was performed to analyse the influence of the treatment, and genotype and their interaction in the different parameter measured in this study. Significance levels: ns = not significant; * P < 0.05; ** P < 0.01; and *** P < 0.001 (n = 5).

|  | K^+^ (mg/g DW) | Na^+^ (mg/g DW) | Ratio K/Na |
| --- | --- | --- | --- |
| Treatment (T) | ns | *** | *** |
| Genotype (G) | *** | ns | *** |
| T x G | *** | *** | *** |

**Table S9.** Ionic contents in quinoa leaves (Ca^2+^, Mg^2+^, P and S). A Two-Way ANOVA followed by a Tukey post-hoc was performed to analyse the influence of the treatment, and genotype and their interaction in the different parameter measured in this study. Significance levels: ns = not significant; * P < 0.05; ** P < 0.01; and *** P < 0.001 (n = 5).

|  | Ca^2+^ (mg/g DW) | Mg^2+^ (mg/g DW) | P (mg/g DW) | S (mg/g DW) |
| --- | --- | --- | --- | --- |
| Treatment (T) | *** | *** | *** | *** |
| Genotype (G) | *** | *** | * | ** |
| T x G | *** | *** | *** | *** |

**Table S10.** Ionic contents in quinoa leaves (Fe^2+^, Mn^2+^, Zn^2+^ and Cu^2+^). A Two-Way ANOVA followed by a Tukey post-hoc was performed to analyse the influence of the treatment, and genotype and their interaction in the different parameter measured in this study. Significance levels: ns = not significant; * P < 0.05; ** P < 0.01; and *** P < 0.001 (n = 5).

|  | Fe^2+^ (µg/g DW) | Mn^2+^ (µg/g DW) | Zn^2+^ (µg/g DW) | Cu^2+^ (µg/g DW) |
| --- | --- | --- | --- | --- |
| Treatment (T) | ns | ns | ns | ns |
| Genotype (G) | ******* | *** | *** | *** |
| T x G | *** | *** | *** | *** |

**Table S11.** Ionic contents in quinoa leaves (Cl^-^, NO_3_**^-^**, PO_4_^3-^ and SO_4_^2-^). A Two-Way ANOVA followed by a Tukey post-hoc was performed to analyse the influence of the treatment, and genotype and their interaction in the different parameter measured in this study. Significance levels: ns = not significant; * P < 0.05; ** P < 0.01; and *** P < 0.001 (n = 5).

**Table S12.** Ionic contents in quinoa leaves (Ctotal, Ntotal, and ratio **C**/N). A Two-Way ANOVA followed by a Tukey post-hoc was performed to analyse the influence of the treatment, and genotype and their interaction in the different parameter measured in this study. Significance levels: ns = not significant; * P < 0.05; ** P < 0.01; and *** P < 0.001 (n = 5).

|  | Ctotal (mg/g DW) | Ntotal (mg/g DW) | Ratio C/N |
| --- | --- | --- | --- |
| Treatment (T) | ns | ns | ns |
| Genotype (G) | *** | * | *** |
| T x G | *** | *** | *** |

|  | Cl^-^ (mg/g DW) | NO_3_^-^ (mg/g DW) | PO_4_^3-^ (m/g DW) | SO_4_^2-^ (mg/g DW) |
| --- | --- | --- | --- | --- |
| Treatment (T) | ******* | ns | *** | ns |
| Genotype (G) | ns | *** | ** | *** |
| T x G | *** | *** | *** | *** |

**Table S13.** N in glycinebetaine, and % of dry weight. A Two-Way ANOVA followed by a Tukey post-hoc was performed to analyse the influence of the treatment, and genotype and their interaction in the different parameter measured in this study. Significance levels: ns = not significant; * P < 0.05; ** P < 0.01; and *** P < 0.001 (n = 6).

|  | N (glycine mg/g DW) | N% (GB) | N (NO_3_^-^ mg/g DW) | N% (NO_3_^-^) |
| --- | --- | --- | --- | --- |
| Treatment (T) | ** | *** | *** | *** |
| Genotype (G) | ns | ns | ** | ** |
| T x G | *** | *** | *** | *** |

**Table S14.** Performance of the PLS-DA model for leaf samples evaluated by 5-fold cross-validation. Classification accuracy, coefficient of determination (R²) and predictive ability (Q²) are shown for models including one to five latent variables.

| Measure | 1comps | 2 comps | 3 comps | 4 comps | 5 comps |
| --- | --- | --- | --- | --- | --- |
| Accuracy | 0.20047 | 0.28275 | 0.44709 | 0.51399 | 0.57786 |
| R^2^ | 0.61065 | 0.82983 | 0.8941 | 0.91255 | 0.92736 |
| Q^2^ | 0.56943 | 0.77742 | 0.80503 | 0.83544 | 0.84066 |

**Table S15.** Performance of the PLS-DA model for seed samples evaluated by 5-fold cross-validation. Classification accuracy, coefficient of determination (R²) and predictive ability (Q²) are shown for models including one to five latent variables.

| Measure | 1comps | 2 comps | 3 comps | 4 comps | 5 comps |
| --- | --- | --- | --- | --- | --- |
| Accuracy | 0.2 | 0.32 | 0.52 | 0.66 | 0.78 |
| R^2^ | 0.73 | 0.84325 | 0.93795 | 0.96158 | 0.96936 |
| Q^2^ | 0.67751 | 0.80781 | 0.90064 | 0.92524 | 0.93484 |

**Table S16.** Amino acids content in quinoa leaves. GABA (γ amino-butyrate); Ala (Alanine); Asn (Asparagine); Asp (Aspartate); Glu (Glutamate); Gln (Glutamine); Gly (Glycine); Ile (Isoleucine); Leu (Leucine); Phe (Phenylalanine); Pro (Proline); Thr (Threonine); Tyr (Tyrosine); Val (Valine). A Two-Way ANOVA followed by a Tukey post-hoc was performed to analyse the influence of the treatment and genotype, and their interaction in the different parameter measured in this study. Significance levels: ns = not significant; * P < 0.05; ** P < 0.01; and *** P < 0.001 (n=5).

|  | GABA | Ala | Asn | Asp | Glu | Gln | Gly | Ile | Leu | Phe | Pro | Thr | Tyr | Val |
| --- | --- | --- | --- | --- | --- | --- | --- | --- | --- | --- | --- | --- | --- | --- |
| Treatment (T) | ns | ** | ns | *** | *** | ** | ns | ns | ns | ns | *** | * | ns | ns |
| Genotype (G) | *** | ns | *** | ns | * | *** | *** | ** | ** | *** | ns | *** | ** | ** |
| T x G | ** | ** | *** | *** | *** | *** | *** | ** | *** | *** | *** | *** | *** | ** |

**Table S17.** Carbohydrates content in quinoa leaves. Fruc (Fructose); Glc (Glucose); Malt (Maltose); Suc (Sucrose). A Two-Way ANOVA followed by a Tukey post-hoc was performed to analyse the influence of the treatment and genotype, and their interaction in the different parameter measured in this study. Significance levels: ns = not significant; * P < 0.05; ** P < 0.01; and *** P < 0.001 (n=5).

|  | Fruc | Glc | Malt | Suc |
| --- | --- | --- | --- | --- |
| Treatment (T) | ** | * | ns | ns |
| Genotype (G) | *** | *** | *** | *** |
| T x G | *** | *** | *** | *** |

**Table S18.** Organic acids content in quinoa leaves. Cit (Citrate); Form (Formate); Fum (Fumarate); Mal (Malate); Succ (Succinate). A Two-Way ANOVA followed by a Tukey post-hoc was performed to analyse the influence of the treatment and genotype, and their interaction in the different parameter measured in this study. Significance levels: ns = not significant; * P < 0.05; ** P < 0.01; and *** P < 0.001 (n=5).

|  | Cit | Form | Fum | Mal | Succ |
| --- | --- | --- | --- | --- | --- |
| Treatment (T) | ns | ns | *** | * | *** |
| Genotype (G) | *** | *** | *** | *** | *** |
| T x G | *** | *** | *** | *** | *** |

**Table S19.** Others metabolites content in quinoa leaves. Bet (Betaine); Cho (Choline); Ea (Ethanolamine); Tri (Trigonelline). A Two-Way ANOVA followed by a Tukey post-hoc was performed to analyse the influence of the treatment and genotype, and their interaction in the different parameter measured in this study. Significance levels: ns = not significant; * P < 0.05; ** P < 0.01; and *** P < 0.001 (n=5).

|  | Bet | Cho | Ea | Tri |
| --- | --- | --- | --- | --- |
| Treatment (T) | *** | ns | ns | ns |
| Genotype (G) | ns | *** | *** | ** |
| T x G | *** | *** | *** | *** |

**Table S20.** Amino acids content in quinoa seeds. GABA (γ amino-butyrate); Ala (Alanine); Asn (Asparagine); Asp (Aspartate); Glu (Glutamate); Gln (Glutamine); Ile (Isoleucine); Leu (Leucine); Phe (Phenylalanine); Pro (Proline); Thr (Threonine); Tyr (Tyrosine); Val (Valine). A Two-Way ANOVA followed by a Tukey post-hoc was performed to analyse the influence of the treatment and genotype, and their interaction in the different parameter measured in this study. Significance levels: ns = not significant; * P < 0.05; ** P < 0.01; and *** P < 0.001 (n=5).

|  | GABA | Ala | Asn | Asp | Glu | Gln | Ile | Leu | Phe | Pro | Thr | Tyr | Val |
| --- | --- | --- | --- | --- | --- | --- | --- | --- | --- | --- | --- | --- | --- |
| Treatment (T) | ns | ns | ns | * | ns | ns | ns | ns | ns | *** | *** | ns | ns |
| Genotype (G) | *** | *** | *** | *** | *** | *** | *** | *** | *** | ** | ns | ns | *** |
| T x G | *** | *** | *** | *** | *** | *** | *** | *** | *** | *** | *** | *** | *** |

**Table S21.** Carbohydrates content in quinoa seeds. Fruc (Fructose); Glc (Glucose); Mlno (Myo-Inositol); Suc (Sucrose). A Two-Way ANOVA followed by a Tukey post-hoc was performed to analyse the influence of the treatment and genotype, and their interaction in the different parameter measured in this study. Significance levels: ns = not significant; * P < 0.05; ** P < 0.01; and *** P < 0.001 (n=5).

|  | Fruc | Glc | MIno | Suc |
| --- | --- | --- | --- | --- |
| Treatment (T) | ns | ** | ns | ns |
| Genotype (G) | *** | *** | *** | *** |
| T x G | *** | *** | *** | *** |

**Table S22. Organic acids content in quinoa seeds.** Cit (Citrate); Form (Formate); Fum (Fumarate); Lac (Lactate); Mal (Malate); Succ (Succinate). A Two-Way ANOVA followed by a Tukey post-hoc was performed to analyse the influence of the treatment and genotype, and their interaction in the different parameter measured in this study. Significance levels: ns = not significant; * P < 0.05; ** P < 0.01; and *** P < 0.001 (n=5).

|  | Cit | Form | Fum | Lac | Mal | Succ |
| --- | --- | --- | --- | --- | --- | --- |
| Treatment (T) | * | ns | ns | ns | ns | *** |
| Genotype (G) | *** | *** | *** | *** | *** | *** |
| T x G | *** | *** | *** | *** | *** | *** |

|  | Bet | Cho | Tri |
| --- | --- | --- | --- |
| Treatment (T) | *** | ns | ns |
| Genotype (G) | *** | * | *** |
| T x G | *** | *** | *** |

**Table S23.** Other metabolites content in quinoa seeds. Bet (Betaine); Cho (Choline); Tri (Trigonelline). A Two-Way ANOVA followed by a Tukey post-hoc was performed to analyse the influence of the treatment and genotype, and their interaction in the different parameter measured in this study. Significance levels: ns = not significant; * P < 0.05; ** P < 0.01; and *** P < 0.001 (n=5).

**SUPPLEMENTARY FIGURES**

**Figure S1.** Effect of salinity on plant height increment in five quinoa genotypes during the experimental period. Plants were grown under control conditions or subjected to 400 mM NaCl. Plant height increment was monitored throughout the salt-treatment period in Puno (PU), Titicaca (TI), Pasankalla (PSK), Vikinga (VK), and Pandela Rosada (PR). Values are means ± SE (n = 15).

**Figure S2.** Permutation tests (1000 permutations) used to validate the PLS-DA models obtained for (A) leaves and (B) seeds. Histograms show the null distribution of the test statistic generated after random permutation of class labels, whereas the red arrow indicates the statistic of the original model. In both datasets, none of the permuted models achieved a test statistic equal to or greater than that of the original model (p < 0.001), confirming the statistical significance and robustness of the PLS-DA models.
